# Geography-Dependent Horizontal Gene Transfer from Vertebrate Predators to Their Prey

**DOI:** 10.1093/molbev/msac052

**Published:** 2022-04-11

**Authors:** Chiaki Kambayashi, Ryosuke Kakehashi, Yusuke Sato, Hideaki Mizuno, Hideyuki Tanabe, Andolalao Rakotoarison, Sven Künzel, Nobuaki Furuno, Kazuhiko Ohshima, Yoshinori Kumazawa, Zoltán T. Nagy, Akira Mori, Allen Allison, Stephen C. Donnellan, Hidetoshi Ota, Masaki Hoso, Tetsuya Yanagida, Hiroshi Sato, Miguel Vences, Atsushi Kurabayashi

**Affiliations:** 1 Faculty of Bio-Science, Nagahama Institute of Bio-Science and Technology, Shiga, Japan; 2 Amphibian Research Center, Hiroshima University, Hiroshima, Japan; 3 Independent Researcher, Yokohama, Japan; 4 School of Advanced Sciences, The Graduate University for Advanced Studies, SOKENDAI, Kanagawa, Japan; 5 Faculté des Sciences, Université d’Antananarivo, Antananarivo, Madagascar; 6 Max Planck Institute for Evolutionary Biology, Plön, Germany; 7 Graduate School of Science, Nagoya City University, Aichi, Japan; 8 Independent Researcher, Berlin, Germany; 9 Graduate School of Science, Kyoto University, Kyoto, Japan; 10 Bernice Pauahi Bishop Museum, Honolulu, HI, USA; 11 South Australian Museum, Adelaide, SA, Australia; 12 Institute of Natural and Environmental Sciences, University of Hyogo, and Museum of Nature and Human Activities, Hyogo, Japan; 13 Graduate School of Advanced Science and Engineering, Waseda University, Tokyo, Japan; 14 Joint Faculty of Veterinary Medicine, Yamaguchi University, Yamaguchi, Japan; 15 Zoological Institute, Braunschweig University of Technology, Braunschweig, Germany; 16 Unit for Environmental Sciences and Management, North-West University, Potchefstroom, South Africa

**Keywords:** horizontal transfer, retrotransposons, biogeography, parasite-dependent transmission, predator and prey

## Abstract

Horizontal transfer (HT) of genes between multicellular animals, once thought to be extremely rare, is being more commonly detected, but its global geographic trend and transfer mechanism have not been investigated. We discovered a unique HT pattern of Bovine-B (BovB) LINE retrotransposons in vertebrates, with a bizarre transfer direction from predators (snakes) to their prey (frogs). At least 54 instances of BovB HT were detected, which we estimate to have occurred across time between 85 and 1.3 Ma. Using comprehensive transcontinental sampling, our study demonstrates that BovB HT is highly prevalent in one geographical region, Madagascar, suggesting important regional differences in the occurrence of HTs. We discovered parasite vectors that may plausibly transmit BovB and found that the proportion of BovB-positive parasites is also high in Madagascar where BovB thus might be physically transported by parasites to diverse vertebrates, potentially including humans. Remarkably, in two frog lineages, BovB HT occurred after migration from a non-HT area (Africa) to the HT hotspot (Madagascar). These results provide a novel perspective on how the prevalence of parasites influences the occurrence of HT in a region, similar to pathogens and their vectors in some endemic diseases.

## Introduction

Horizontal transfer (HT) is a passage of genetic material between organisms through a mechanism other than reproduction. HT is well known in prokaryotes, where it often serves as a driving force for evolution by modifying genome structure, gene content, and/or gene expression pattern in the host genomes (Soucy et al. 2015). Whereas HT has rarely been documented among eukaryotes (Anderson 2005), recent evidence suggests that HTs among multicellular organisms are more common than previously thought (Huang 2013) and that the majority of these HTs correspond to transfers of transposable elements (TEs) (Schaack et al. 2010). Owing to the intrinsic ability to transpose within genomes and expand their copy numbers, TEs are the most abundant component of many eukaryotic genomes (e.g., Lander et al. 2001; Schnable et al. 2009). It is also known that TEs are mutagens of the genome and occasionally cause genetic disorders (e.g., Bourque et al. 2018). Thus, understanding the trends of occurrence and mechanisms of transmission of TE HTs is important to understand how potential alien mutagens are acquired from other organisms, how they affect genome evolution, and how they impact the fitness of the host organisms (Kazazian and Moran 2017).

Recent large-scale genome analyses have detected a number of HTs of TEs even among multicellular animals (Walsh et al. 2013; Suh et al. 2016; Ivancevic et al. 2018; Zhang et al. 2020). Genomic surveys across insect taxa have shown a tendency of phylogenetic relatedness and geographical proximity favoring HTs (Peccoud et al. 2017). It has also been suggested that host–parasite interactions facilitate HTs and that parasites may mediate HTs among vertebrates, by the finding of TEs transferred among vertebrates by blood-sucking triatomine bugs, ticks, and endoparasitic nematodes (Gilbert et al. 2010; Walsh et al. 2013; Dunemann and Wasmuth 2019). However, since genome-based analyses to date are largely limited by the extent of geographic and taxon sampling, geographic trends and transmission modes of intermetazoan HT propagation are poorly understood.

Bovine-B (BovB) is a unique LINE retrotransposon initially identified in cattle (*Bos*) (Szemraj et al. 1995) and makes up more than 18% of its genome (Walsh et al. 2013). Subsequent studies have shown that BovB is vertically transmitted in squamates (snakes and lizards) and that BovB in ruminants originated by HT from snakes (Kordis and Gubensek 1998). Moreover, it has recently been shown that BovB is horizontally transmitted among a wider range of metazoans (Walsh et al. 2013; Ivancevic et al. 2018).

Given the prevalence of amphibians in the diet of snakes (Colston et al. 2010), we hypothesized that BovB HT might also occur between snakes and frogs. Remarkably, our preliminary analyses revealed that the genomes of several Malagasy frogs contain a large number of BovB sequences having quite high nucleotide similarity with those of snakes. The discovery led to the following questions, addressed herein: (1) in which direction did the BovB HT occur (from frog to snake or from snake to frog)? (2) Is the BovB HT between snakes and frogs an isolated (only in Madagascar) or worldwide phenomenon? (3) In what mode did this HT occur, that is, by direct contact or by the mediation of vector organisms?

We addressed the aforementioned three questions using a transcontinental and comprehensive taxon sampling comprising 106, 149, and 42 species of snakes, frogs, and their parasites, respectively. We further aimed to understand geographical trends in the frequency of occurrence of intervertebrate HTs which to date has remained poorly studied. Our analyses showed that, counterintuitively, BovBs in frogs were derived from those in snakes, indicating recurrent HTs of BovBs from predators (snakes) to their prey (frogs). Furthermore, we demonstrate that there is clear geographical variation in the frequency of occurrence of horizontal transmission of BovB, with Madagascar being a hotspot, which may be mediated by the presence of BovB-carrying parasites in the region. Our study provides initial and novel perspectives on the global scale regional pattern of the occurrence of horizontal transmission and suggests that intervertebrate HT has a transmission mode analogous to the infection mode of some vector transmitted endemic diseases, such as malaria (Phillips et al. 2017).

## Results and Discussion

### Detection of BovB from Frog Genomes

Our initial screening detected BovB fragments highly similar to those previously found in vipers (sequence similarity >94%) in the genomes of three frog species from Madagascar (supplementary fig. S1 and table S1, Supplementary material online). To investigate the phylogenetic diversity of the taxa involved in HT, and the geographical distribution of BovB-positive frogs, we performed PCR screening of 109 reptile species including 20 of the 30 snake families and three lizard families, and 152 amphibian species from 28 of the 56 frog families (supplementary data 1, Supplementary Material online). BovB PCR products were amplified in all reptiles and 50 frog species (34%). The ratio of BovB-positive frogs varied by geographic region and was highest in Madagascar (91%) (Fisher’s exact test with Holm correction, *P* < 0.05; table 1). To rule out false positives due to amplification of DNA contamination, we performed dot blot analysis and detected intense signals in BovB-positive species (supplementary figs. S2 and S3, Supplementary Material online). Fluorescence in situ hybridization analysis with snake BovB sequence as the probe showed clear BovB signals on the frog chromosomes and nuclei (supplementary fig. S4, Supplementary Material online). We furthermore confirmed BovB sequences in six genome assemblies corresponding to BovB-positive frog species, among 21 amphibians for which whole-genome assemblies were available as of 2021 (supplementary table S1, Supplementary Material online). These results confirm that the BovB detected in frogs did not originate from field sampling or laboratory or database contamination. Partial genome sequencing and dot blot analysis also revealed that BovB sequences are abundant, contributing up to 0.53% of the frog genomes (supplementary table S1 and data S2, Supplementary Material online).

**Table 1.** Frequency of BovB-Positive Frog and Parasite Species in Each Region and Comparisons With Those in Madagascar.

Region	Frog		Parasite	
	Percentage	*P*-value (vs. Madagascar)	Percentage	*P*-value (vs. Madagascar)
Madagascar	91% (29/32)	—	50% (4/8)	—
Western–Central–Southern Asia	50% (3/6)	0.039	—	—
Oceania	44% (4/9)	0.021	—	—
Europe	40% (2/5)	0.045	—	—
Southeast Asia	26% (5/19)	1.6 × 10^−5^	—	—
East Asia	23% (7/30)	4.3 × 10^−7^	2.9% (1/34)	2.9 × 10^−3^
Africa	0% (0/18)	5.2 × 10^−10^	—	—
North America	0% (0/9)	3.1 × 10^−6^	—	—
Central–South America	0% (0/21)	5.1 × 10^−11^	—	—
Average	34% (50/149)	—	12% (5/42)	—

Raw numbers of species used to calculate percentages are in parentheses. *P*-value was adjusted using Holm correction.

### Distribution and Timing of BovB Invasions

**Fig. 1.**
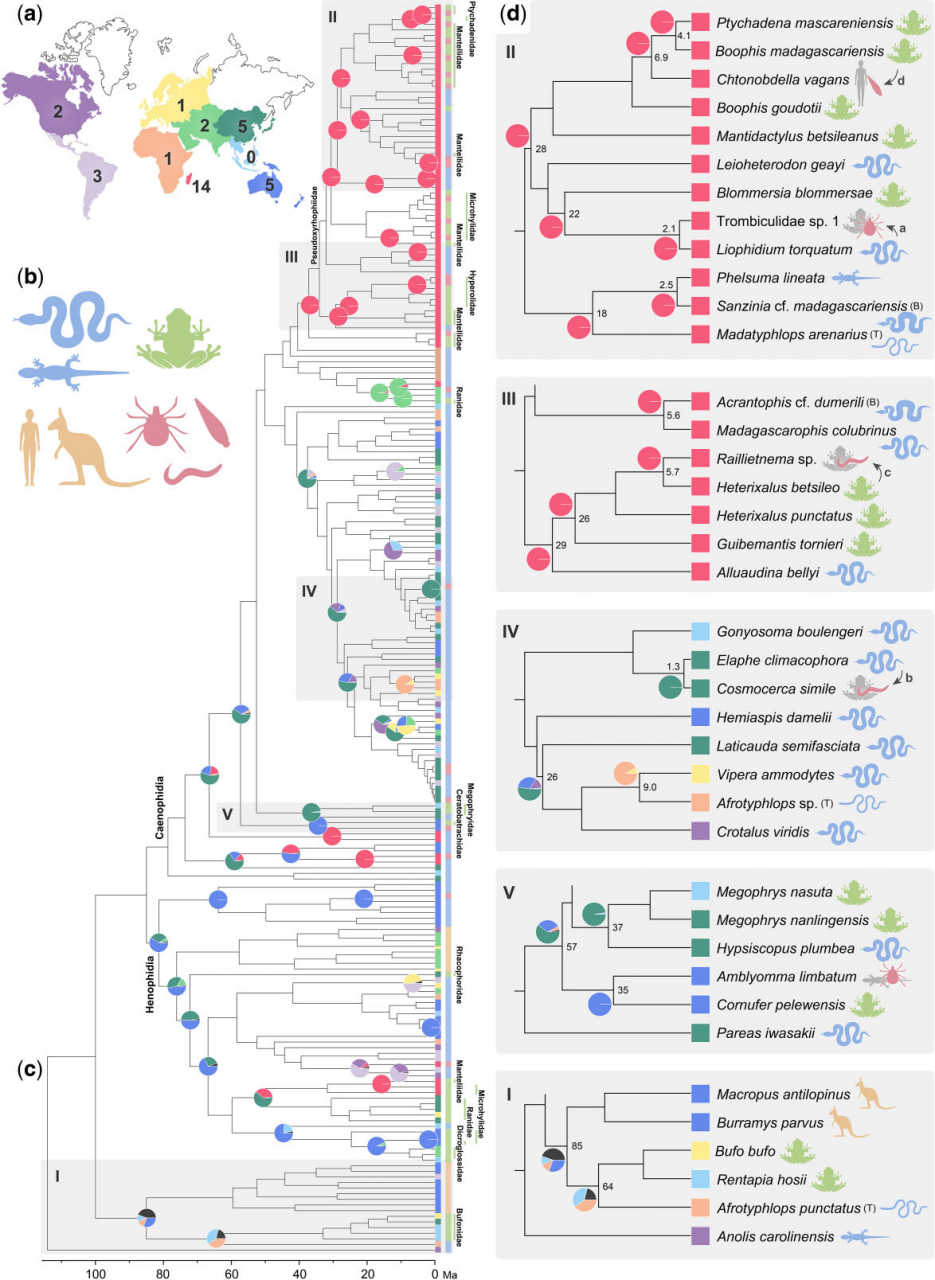
Phylogeny, ages, and geographic distribution of HTs of squamate-type BovB. (*a*) The nine biogeographic areas defined in this study. Numbers at each region on the map indicate the numbers of HTs among reptiles and amphibians occurring within the past 50 Ma (33 of total 54 HTs). (*b*) The animal taxa surveyed (reptile, blue; frog, green; mammal, orange; and parasite, red). (*c*) The time tree of BovBs from 222 OTUs with HT occurrence geographic region estimated by BioGeoBEARS. Each tip of the tree is color-coded according to distribution within nine world regions (left, the color code is the same with *a*) and taxa (right = *b*). Pie charts on nodes represent the relative probabilities of occurrence areas for the 54 possible HTs. Reconstructions resulting in more than two possible regions are shown in dark gray. The compartments marked with Roman numerals correspond to those in *d*. (*d*) The topologies show remarkable HTs. Numbers at nodes indicate divergence time (Ma). The blindsnakes and Malagasy boas are labeled by capital letters in parentheses (T—Typhlopidae and B—Boidae, respectively). The gray-colored animal symbols represent the hosts of BovB-positive parasites. The arrows with small letters correspond to those in figure 2.

A phylogenetic analysis of 211 new BovB consensus sequences obtained via single-molecule real-time (SMRT) sequencing (Eid et al. 2009), and 74 known BovBs, yielded a phylogenetic tree largely concordant with the ortholog-based phylogenetic relationships at the family level for the host squamates (Zheng and Wiens 2016) (fig. 1*c*, supplementary fig. S5, Supplementary Material online). Several instances where BovBs from distantly related taxa clustered together imply the occurrence of HT among squamates (e.g., many blindsnakes and Malagasy boas; fig. 1*d*-II–IV, T and B). A reduced data set with 222 operational taxonomic unit (OTUs) (removing 31 of the nonsquamate-type BovBs; supplementary figs. S6 and S7, Supplementary Material online), confirms the congruence of the squamate BovB tree with the general host squamate phylogenetic relationships at species, genus, and family levels (tested by *I*_cong_ index [de Vienne et al. 2007] and Triples metric [Dobson 1975]; see Materials and Methods and supplementary table S2, Supplementary Material online). This suggests that BovBs were primarily transmitted via vertical inheritance in squamates. Some topological consistency was also detected between the BovB tree and established phylogenetic relationships of frogs but with a lower degree of concordance than in squamates (supplementary table S2, Supplementary Material online). This result is consistent with the hypothesis that the BovBs of frogs were only rarely acquired by vertical transmission but rather transmitted from snakes via multiple HT events with a unique transfer direction, from predators to their prey.

To infer the timing of HT events, we then time-calibrated the BovB maximum likelihood (ML) tree (supplementary fig. S8, Supplementary Material online). In the 222-OTUs chronogram (fig. 1*c*), the nodes at which different families diverged and the corresponding branch topology differed from the general phylogenetic consensus (Zheng and Wiens 2016; Feng et al. 2017; Hime et al. 2021) were identified as HT points. This procedure led to the identification of at least 54 probable instances of HTs, occurring across time between 1.3 and 85 Ma.

In the 222-OTUs tree, frog BovBs were found in various clades (fig. 1*c*). The BovBs of the frog family Bufonidae were monophyletic and closely related to that of *Afrotyphlops punctatus*, a representative of the blindsnakes that form the sister group of all other snakes (fig. 1*d*-I). This observation indicates that a BovB HT occurred from an early diverged snake group to the common ancestor of bufonid frogs around 64 Ma and that BovB has since been vertically transmitted to the modern bufonid lineages. The clade that comprised primarily the snake superfamily Henophidia also contained BovBs of 15 frog species from five families: Dicroglossidae, Mantellidae, Microhylidae, Ranidae, and Rhacophoridae (fig. 1*c*). In another major clade, comprising mainly the superfamily Caenophidia, we observed BovBs of 30 frog species from seven families (Ceratobatrachidae, Hyperoliidae, Mantellidae, Megophryidae, Microhylidae, Ptychadenidae, and Ranidae); and one Malagasy caenophidian subclade alone, the Pseudoxyrhophiidae, contained BovBs of 26 frog species from four families (Hyperoliidae, Mantellidae, Microhylidae, and Ptychadenidae) (fig. 1*c*).

There were over 10 snakes in which the pattern of BovBs did not reflect the host phylogeny. In seven blindsnakes of the superfamily Typhlopoidea, the BovBs identified were derived from different snake BovB lineages. In addition, two Malagasy boas, which should harbor BovBs inherited from the Henophidia clade, had BovBs from Malagasy caenophidians (the family Pseudoxyrhophiidae) (fig. 1*d*-II, III). These cases are probably due to snake-to-snake horizontal transmission, in which a newly integrated BovB has become dominant over the original BovB. Although it is assumed that the BovBs of marsupials are closely related to those of some squamates or ruminants (Walsh et al. 2013; Ivancevic et al. 2018), our phylogeny based on more detailed sampling showed that BovBs in the marsupials form a monophyletic group with those of a snake, *A. punctatus*, and bufonid frogs (fig. 1*d*-I). This indicates that the origin of BovBs in marsupials is more complex than previously thought. We identified several more notable instances of HTs, details of which are described in the supplementary note, Supplementary Material online.

### Potential Parasite Vectors Transferring BovB

Whereas the phylogenetic analysis strongly suggests a high proportion of BovB HTs from snakes to frogs, it does not clarify whether the transmission occurred directly or via vector organisms. We, therefore, extended our investigation to parasites that could constitute potential vectors of the horizontal transmission of BovBs. A total of 97 individuals of 42 parasite species associated with reptiles and amphibians (supplementary data 1, Supplementary Material online) were collected from Madagascar and East Asia, two regions with different proportions of BovB-positive frogs (91% vs. 23%). These include endoparasites such as nematodes, trematodes, acanthocephalans, frog-specific intracutaneous mites, as well as more generalist ectoparasites such as leeches and mosquitos. Our screening identified BovB PCR products in parasites from three phyla, Annelida, Arthropoda, and Nematoda. To exclude the possibility of DNA contamination from the vertebrate hosts of these parasites, DNA extractions from parasites were performed after eliminating as much of the digestive tract as possible, and an event was not considered to be a HT when the parasite and host BovBs showed more than 98% similarity.

The BovBs from the invertebrate parasites were commonly derived from non-host snake or frog lineages. The BovB of a chigger, Trombiculidae sp. 1, isolated from a mantellid frog, *Blommersia blommersae*, from Madagascar was more closely related to that of a Malagasy snake, *Liophidium torquatum* (sequence similarity 99.8%), than that of its host (96.1%) (figs. 1*d*-II and 2*a*). Similarly, the BovB of a nematode, *Cosmocerca simile*, isolated from the BovB-negative Japanese rhacophorid frog, *Buergeria japonica*, was included in the clade of the snake family Colubridae and was most closely related to a species also occurring in Japan, *Elaphe climacophora* (sequence similarity 100%) (figs. 1*d*-IV and 2*b*). These two parasites are thus plausible vectors that may have transferred the snake-type BovBs to frogs by moving between hosts. The BovB of a tick, *Amblyomma limbatum*, collected from an Oceanian lizard, *Tiliqua rugosa* (reported in Walsh et al. 2013), was monophyletic with that of a ceratobatrachid frog, *Cornufer pelewensis*, also from Oceania (fig. 1*d*-V). This tick species was probably involved in another BovB HT from reptiles to frogs. The BovBs of the Malagasy nematodes isolated from the mantellid frogs *Mantidactylus* (*Md*.) *femoralis* and *Md. betsileanus* emerged in the clade of the frog family Hyperoliidae (figs. 1*d*-III and 2*c*), an observation indicating that BovB transfer by parasites may also occur among frogs. A BovB sequence similar to that from the mantellid frog, *Boophis madagascariensis*, was detected in a Malagasy leech, *Chtonobdella vagans*, collected from humans (figs. 1*d*-II and 2*d*). This observation suggests that there is potential for physical transfer of BovB among a wide range of vertebrate taxa in Madagascar. Although it is not known whether these parasite taxa can move between snakes and frogs (supplementary data 1, Supplementary Material online), the BovB transmission via these parasites can be explained if (1) they have wider host ranges than previously known, (2) their ancestors parasitized both snakes and frogs, and/or (3) host switching (from reptiles to frogs) occurred in the parasite ancestral lineages. The percentage of parasite species collected in Madagascar with a version of BovB different from that of their host (sequence similarity <98%) and not clearly due to contamination (50%) was significantly higher than that in Japan (2.9%) (Fisher’s exact test, *P* < 0.01; table 1).

**Fig. 2.**
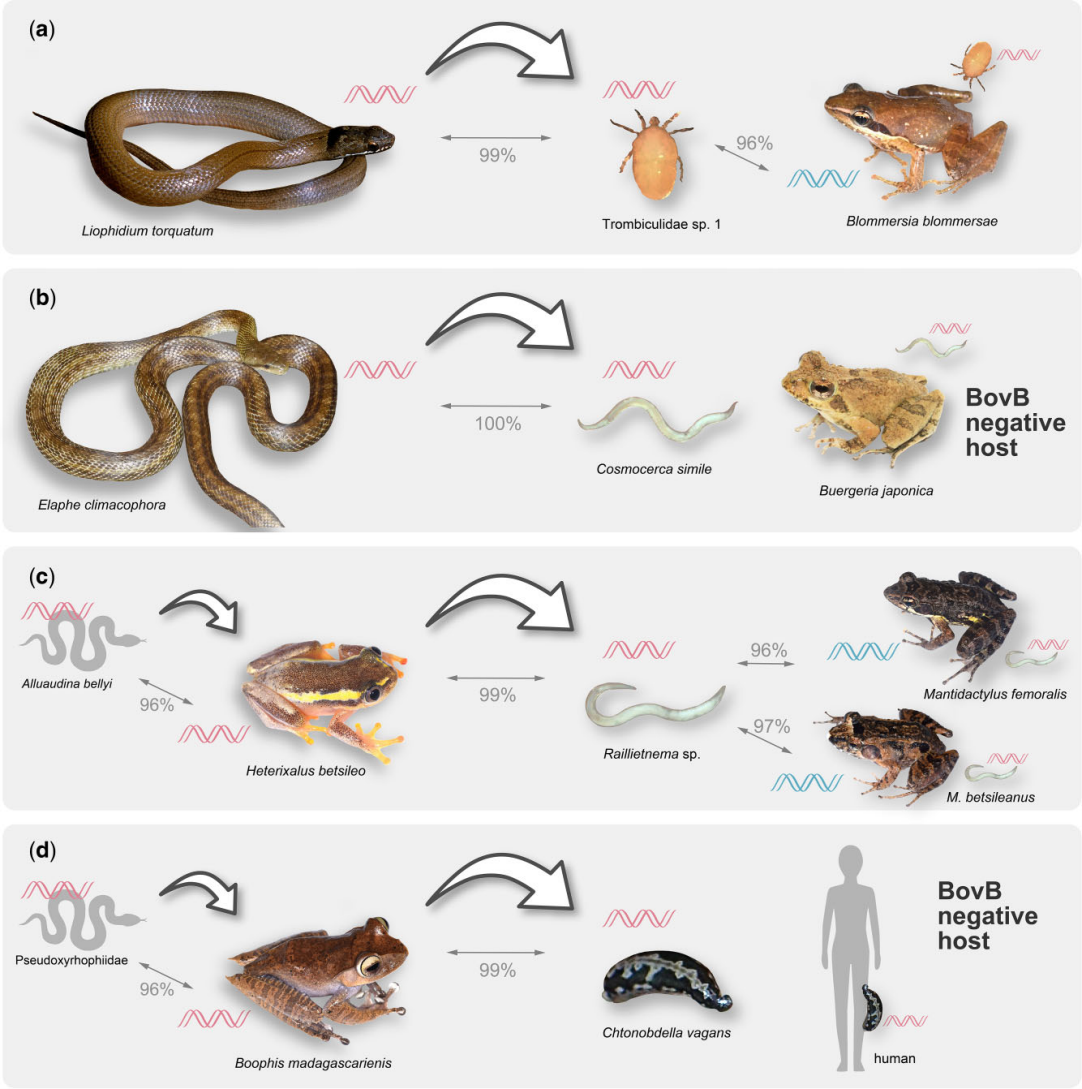
Transmission pathways of snake BovBs via parasites. The representatives of HTs of snake BovBs via parasites are shown. The thick and thin arrows show the direction of HT and the similarities of BovB sequences between taxa, respectively.

### Madagascar as a Hotspot of BovB Horizontal Transfer

The result of geographical area reconstruction for the 54 HTs estimated here revealed their widespread occurrence across the globe (supplementary fig. S9, Supplementary Material online). Focusing on those 33 HTs inferred among squamates and amphibians over the past 50 million years, that is, the time after the current continental arrangement formed with the collision of India and Eurasia (Meng et al. 2012) confirmed an uneven geographical distribution. The number of HT events occurring within the past 50 Ma was particularly high in Madagascar with 14 HTs (fig. 1*a*) and lower in East Asia and Oceania with five each, and Africa with one HT. In Madagascar, the ratio of the number of HTs to the number of species was significantly higher than the ratio in the other six regions, except in Oceania and Western–Central–Southern Asia (binominal test with Holm correction, *P* < 0.05). However, the HT frequencies in the latter regions were probably overestimated compared with Madagascar, where many samples were taken within genera in which the inheritance of BovB is vertical. We compared the ratio of the number of HTs to the number of genera and found that the HT frequency in Madagascar was significantly higher than that in any other region (supplementary table S3, Supplementary Material online). Most of the Madagascan biota comprises descendants from African origin dispersers (Yoder and Nowak 2006), and in frogs, two lineages represented by *Heterixalus* and *Ptychadena mascareniensis*, are known to have migrated from Africa to Madagascar 19–30 and 9.8–22.7 Ma, respectively (Vences et al. 2003; Zimkus et al. 2017). In the course of our geographical analyses, it was shown that the BovB HTs to these frogs occurred in Madagascar. This result is supported by the consistency of the estimated ages of HT events with those of their dispersal and the fact that the closely related frogs indigenous to Africa, *Hyperolius* and *Ptychadena nilotica*, are BovB-negative according to PCR. These examples of BovB HTs in postmigrant Madagascan frogs exemplify their region-specific occurrence, analogous to malaria infection in humans who migrated to malaria-afflicted areas.

Although TEs in genomes are generally transcriptionally silenced by epigenetic regulation (Slotkin and Martienssen 2007), we found evidence that BovB may be transcribed both in snakes and frogs. Novel RNA-seq data contained BovB fragments in three snakes (*Madatyphlops* sp., *Mimophis mahfalensis*, and *Thamnosophis lateralis*) and seven frogs (*Aglyptodactylus madagascariensis*, *Boophis tephraeomystax*, *Mantella* [*Mt.*] *expectata*, *Mt. laevigata*, *Md. betsileanus*, *Md. multiplicatus*, and *Plethodontohyla notosticta*) collected from Madagascar but not in a BovB-negative frog from Southeast Asia (*Polypedates otilophus*). In particular, almost full-length BovB sequences with long open reading frames were assembled from two Malagasy snakes (*M. mahfalensis* and *T. lateralis*).

Whereas the presence of BovB in a diverse array of parasites leads us to favor the hypothesis of parasite-mediated transmission, the hypothesis of direct transmission remains viable. In fact, one case of BovB HT from a frog lineage to a frog-eating snake lineage was detected in our phylogenetic analyses (the BovB of Indomalayan xenopeltid snake is nested in the BovB clade of Indomalayan/Australasian ranoid frogs: supplementary note, Supplementary Material online), and this finding appears to be the first evidence of direct horizontal transmission of retrotransposon between vertebrates via predation. On the other hand, a scenario of regularly occurring direct HTs from snakes to frogs would require invoking failure of predation attempts, where the escaped prey acquired BovB through direct contact and injury. However, unlike with hard-bodied prey such as many squamates, snake teeth can easily penetrate the soft unprotected body of frogs to secure a firm grip. Although comprehensive studies on the topic are rare, it appears unlikely that frogs would commonly escape and survive after a successful snake bite (but see Costa and Trevelin 2020). Furthermore, based on SquamataBase (Grundler 2020), the proportion of snakes known to consume frogs does not differ significantly among regions (*χ*^2^ test with Holm correction, *P* > 0.05; supplementary table S4, Supplementary Material online), suggesting that direct transmission of BovB does not contribute substantially to the region-specific frequency of BovB HTs that we have observed.

## Conclusions

In our study, we analyzed the HT of BovB from snakes to frogs using a comprehensive sampling of snakes, frogs, and their parasites. Our results suggest that Madagascar is a hot spot for BovB HTs, in which a variety of parasites mediate HT through host-to-host movement. The observations that leeches infesting humans possess frog-type BovB and that BovB may be expressed in Malagasy snakes and frogs suggest that the BovB HTs may occur among an even wider range of vertebrate taxa in Madagascar. Our finding of these extensive BovB HTs mediated by parasites provides a mechanism for the rapid and broad taxonomic transmission of genetic elements. We also showed that BovB, which originated in frogs by HT from snakes only 27.7 Ma, has accumulated, to constitute 0.53% of the genome of the mantellid frog, *Boophis goudotii* (fig. 1*d*-II, supplementary table S1, Supplementary Material online). Mammals are known to be highly susceptible to BovBs introduced from snakes 76–85 Ma, which account for 1.3% of the genome in opossum, 15.2% in sheep, and 18.4% in cattle (Walsh et al. 2013). The worldwide occurrence of BovB HTs revealed in our study highlights the potential for genomic modifications by alien TEs in more diverse vertebrate taxa than previously conceived. In the future, BovB may occupy a position by its proliferation in a genome of host vertebrates similar to L1-LINE, the partner of Alu elements, which comprise 17% of the human genome (Lander et al. 2001).

In this study, we confirmed the proliferation of BovB copies in the frog chromosomes of HT destination and also the BovB clades of closely related frogs (supplementary fig. S9, Supplementary Material online), suggesting that BovB was integrated in the germline of their ancestors and is then passed on to their descendants. On the other hand, regarding the parasites, our data do not allow discriminating between two scenarios: (1) the BovBs could be integrated into the parasite genomes and then passed on to their hosts (biological transmission) or (2) the parasites may just carry bacteria and/or viruses whose genomes contain BovB, or cells of a previous BovB-containing host (e.g., blood cells derived from blood-sucking: mechanical transmission). Further studies including genome sequencing and fluorescence in situ hybridization (FISH) analysis of parasites and metagenomic analyses of bacteria and viruses infecting snakes and frogs will shed light on the detailed transmission mode of intervertebrate HT mediated by parasites, and on the germline integration of BovB in the host organisms.

## Materials and Methods

### Sampling of Reptiles, Amphibians, and Parasites

We sampled 121 individuals of 109 reptile species from 20 snake and three lizard families and 167 individuals of 152 amphibian species from 28 frog, two salamander and one caecilian families. A total of 97 parasite specimens from five animal phyla (Acanthocephala, Annelida, Arthropoda, Nematoda, and Platyhelminthes) were collected from reptiles and amphibians in Madagascar and Japan (supplementary data 1, Supplementary Material online). Total DNA was extracted from frozen or ethanol-preserved liver or muscle tissues of reptiles and amphibians, using phenol/chloroform extraction. For parasites, total DNA was extracted from muscles, excluding the digestive tract as much as possible, to rule out possible contamination from ingested host tissue. Most of the tissue specimens used were from the museum and personal zoological collections but we also took parasites from live frogs and snakes. In addition, we used tissue and cell specimens from live snakes and frogs for RNA sequencing and FISH experiments. This research was conducted in Madagascar under collection and exportation permits for snakes, frogs, and parasites issued by the Malagasy authority (No. 215/16-MEEF/SG/DSAP/SCB.Re and 010N-EA01/MG17, respectively). The experiments with live vertebrates were performed under the permissions from the Ethics Committees for Animal Experiments of Hiroshima University (# C16-22 and G17-1) and Nagahama Institute of Bio-Science (# 085).

### Species Identification and Elimination of Contamination

The identification of the species of reptiles, amphibians, and parasites used in this study was confirmed by analysis of partial sequences of mitochondrial cytochrome apoenzyme b (Cytb) in reptiles, 16S rRNA in amphibians, and nuclear 18S rRNA in parasites. Each fragment was amplified using specific primers (Kocher et al. 1989; Bossuyt and Milinkovitch 2000; Burbrink et al. 2000) (supplementary table S5, Supplementary Material online) and EmeraldAmp PCR Master Mix (Takara Bio Inc., Shiga, Japan). PCR was conducted using the following temperature cycling: initial denaturation at 94 °C for 5 min, followed by 35 cycles of denaturation at 94 °C for 30 s, annealing between 46 and 55 °C based on gene-specific gradients for 30 s, and elongation at 72 °C for 1 min, ending with a 7 min elongation step at 72 °C. Sequencing of the amplified products was performed using BigDye Terminator v. 3.1 Cycle Sequencing Kits (Applied Biosystems, Foster City, CA). The sequencing reactions were ethanol precipitated and run on an ABI 3100xl automated DNA sequencer (Applied Biosystems). Sequence electropherograms were checked using MEGA X (Kumar et al. 2018), and samples with double peaks were excluded to remove potential contamination. The species were identified using Megablast search (Zhang et al. 2000) of the National Center for Biotechnology Information (NCBI) databases.

For reptiles, the specimen was identified as the top matched species if the maximum identity was 95% or more. When the maximum identity fell in the range 90–94%, and Cytb sequence data for the morphologically identified species were not available from the NCBI, the morphologically identified name was labeled with “cf.”. When the data of the morphologically identified species were available from the NCBI, the top matched species in the BLAST search was labeled with “cf.”. When the maximum identity was 85–89% or less than 85%, “sp.” was labeled in the genus or family of the top matched species. The scientific name was assigned according to Uetz et al. (2020).

For amphibians, the specimen was identified as the top matched species when the identity was 97% or over, whereas “sp.” was added to the top genus match when the identity was less than 97%. The scientific name was assigned according to Frost (2020). For parasites, the specimen was identified as the top species match when the identity was 99.5% or more. When the identity was less than 99.5%, “sp.” was added to the taxonomic rank common to the highly matched species.

### PCR Screening and Multiplex Sequencing

PCR screening for the presence of BovB was conducted using 24 primer combinations, using four forward and six reverse primers (supplementary table S5, Supplementary Material online). BovB fragments were amplified by standard and touch-down PCR methods using LA taq Hot Start Version (Takara Bio). The temperature cycling of PCR was as follows: for standard PCR, 2 min at 94 °C followed by 37 cycles at 94 °C for 25 s, 57.5 °C for 30 s, 68 °C for 3 min, and 72 °C for 4 min, and for touch-down PCR, 2 min at 94 °C followed by six cycles at 94 °C for 25 s, 65 °C for 30 s, and 68 °C for 3 min; six cycles at 94 °C for 25 s, 62.5 °C for 30 s, and 68 °C for 3 min; and 25 cycles at 94 °C for 25 s, 60 °C for 30 s, 68 °C for 3 min, and 72 °C for 4 min. For the samples in which the candidate band of BovB was amplified, PCR was repeated with primers containing 16 bp barcode sequences. The candidate fragments were purified using gel extraction with Qiaex II Gel Extraction Kits (Qiagen, Hilden, Germany), and 120 ng of DNA was collected for each sample. To prevent DNA contamination, the PCR and gel extraction steps were carried out by different people for reptiles, amphibians, and parasites. Because BovB has a multi-locus nature, the PCR fragments amplified from multiple loci are mixed together. Thus normal Sanger sequencing approaches could not be applied for sequencing the PCR products. To sequence the single BovB fragment derived from a single locus from the PCR fragment, we used SMRT technique (Eid et al. 2009). The PCR fragments obtained were pooled in a single tube and sequenced in 13 runs of Multiplex-Amplicon analyses using PacBio RS II (Pacific Bioscience, Menlo Park, CA). We outsourced the library construction and sequencing to the Center of Medical Innovation and Translational Research of Osaka University, Duke GCB (Sequencing and Genomic Technologies of Duke University), Integral Inc. (Tokyo, Japan), Macrogen Japan Corp. (Tokyo, Japan), and Tomy Digital Biology Inc. (Tokyo, Japan). The raw sequence data generated by PacBio RS II sequencing were assembled in circular consensus sequences (CCSs), and the CCSs were demultiplexed according to their PCR barcodes using the SMRT Portal (Pacific Biosciences). The CCS reads were selected based on the sequence qualities (quality value ≥ 30), and then a consensus sequence was constructed for each sample with over 50 clean reads. The parameters of the filtering were as follows: (1) contain primer sequences on both ends; (2) average quality score is over 99%; (3) more than 70% sequence similarity with BovB_VA [one of the full-length consensus sequences of BovB in snakes (Zupunski et al. 2001) (supplementary fig. S1, Supplementary Material online) showing the highest nucleotide similarities with the BovBs from Malagasy frogs among the snake BovBs reported so far]; and (4) length falls in the range 1,300–3,500 bp. The original programs to perform this filtering process are available on GitHub (https://github.com/mizuno-hideaki/horizontal-gene-transfer).

### Molecular Phylogenetic Analyses

We added 74 known BovB sequences (Bao et al. 2015; Ivancevic et al. 2018) to the data from 211 specimens consisting of 121 reptile individuals of 109 species of 100 genera of 23 families, 65 frog individuals of 50 species of 30 genera of 10 families, and 25 parasite individuals of three phyla newly obtained in this study. These 285 BovB sequences were aligned using MAFFT with the L-INS-I option (Katoh et al. 2019), and a preliminary ML phylogenetic analysis was conducted using RAxML v. 8.2.10 with the rapid hill-climbing algorithm (Stamatakis 2014). The best substitution model was estimated using Kakusan4 (Tanabe 2011), and the independent general time-reversible + gamma distribution (GTR + G) substitution model was applied. The supports for the internal branches of reconstructed trees were evaluated using bootstrap percentages calculated with 1,000 pseudoreplicates. Based on the result, we eliminated the nonsquamate-type BovB sequences (31 sequences more primitive than the BovB of the anole lizard, *Anolis carolinensis*) and rebuilt a BovB data set that appears to be vertically transmitted in squamates to perform precise divergence time estimation and ancestral range estimation of the BovB sequences derived from snakes. We also deleted 32 sequences that are almost identical within species. The 222-OTUs data set was aligned by MAFFT, and phylogenetic trees were inferred using ML and Bayesian inference (BI). In these analyses, the GTR + G model was selected as the best substitution model by Kakusan4. The ML phylogeny was inferred using RAxML as described previously. The BI analysis was performed with MrBayes v. 3.2.6 (Ronquist et al. 2012). Two independent runs of four Markov chains were conducted for 20 million generations, and the tree was sampled every 1,000 generations. The convergence of the posterior distribution of model parameters (all parameters reached 200) was checked using Tracer v. 1.7.1 (Rambaut et al. 2018), and the first 10% of samples were discarded as burn-in. The supports for the internal branches were evaluated using Bayesian posterior probabilities. The tree topologies inferred by ML and BI were almost identical with some differences in the branching pattern and the presence of unresolved nodes (polytomies) in the BI tree (supplementary figs. S6 and S7 and table S6, Supplementary Material online). To verify the vertical transmission of BovB in squamates, *I*_cong_ index (de Vienne et al. 2007) and normalized Triples metric (Dobson 1975) between the topologies of squamates on the 222-OTUs ML tree and a tree constructed based on orthologous genes (Pyron et al. 2013) were calculated. They were assessed at the species, genus, and family levels, and compared with the values among frog topologies (Pyron and Wiens 2011) (supplementary table S2, Supplementary Material online). In the 222-OTUs ML tree (fig. 1*c*), we recognized the nodes at which phylogenetically distant taxa (above familial level) diverged and the corresponding topology is obviously different from the general phylogenetic hypothesis (Pyron and Wiens 2011; Pyron et al. 2013) as the HT points.

### Divergence Time Estimation

A Bayesian relaxed-clock analysis was performed using BEAST v. 2.6.0 (Bouckaert et al. 2019) to date the occurrence of HT events. The 222-OTUs ML topology was used as the reference tree. We applied the Yule process (Yule 1924) to describe cladogenesis. Markov chain Monte Carlo chains were run for one billion generations with one sampling per every 1,000 generations, and the first 10% of generations were discarded as burn-in. The posterior distributions of the model parameters were checked in the same way as the BI analysis above. Based on the TimeTree database (Kumar et al. 2017), we employed eight calibration points: (1) Henophidia–Caenophidia split, 72–92 Ma; (2) Xenodermidae–Pareidae split, 59–89 Ma; (3) most recent common ancestor (MRCA) of Boinae, 43–68 Ma; (4) Loxocemidae–Pythonidae split, 30–68 Ma; (5) MRCA of Lamprophiidae, Prosymnidae, Psammophiidae, Pseudaspididae, and Pseudoxyrhophiidae, 37–51 Ma; (6) MRCA of *Heterixalus*, 15.6–25.7 Ma; (7) MRCA of *Thamnosophis*, 8.06–23.84 Ma; and (8) MRCA of *Bufo*, 11.1–17.8 Ma.

### Geographical Area Estimation

We defined nine biogeographic areas (Africa, Europe, Madagascar, Oceania, North America, Central–South America, East Asia, Southeast Asia, and Western–Central–Southern Asia). Each reptile, amphibian, and mammal species was assigned to a location, based on current distribution data from The Reptile Database (Uetz et al. 2020), Amphibian Species of the World (Frost 2020), and Mammal Species of the World (Wilson and Reeder 2005), respectively. For the globally distributed species such as domestic livestock or invasive species, the estimated origins were assigned (Pidancier et al. 2006; Rezaei et al. 2010; Yindee et al. 2010; Pyron and Wallach 2014; Upadhyay et al. 2017; Krinsky 2018), and the collection areas were used for parasites. Ancestral range reconstruction was performed using BioGeoBEARS (Matzke 2013). The maximum number of ancestral areas allowed at each node was set to four. We compared all six models implemented in BioGeoBEARS. The AIC selected the DIVALIKE + J as the best fitting model, and this was subsequently used to infer the most likely geographic history of BovB HT events (supplementary table S7, Supplementary Material online). The availability of connections between areas was unconstrained. The ancestral region with the highest probability at the node at which HT was estimated to have occurred was considered to be the region of occurrence.

### mRNA Sequencing

Tissues from each sample were preserved in RNAlater and frozen at −80 °C. Total RNA extraction was carried out using a standard trizol protocol from about 20–100 mg of tissue per specimen (combined or separate skin, muscle, or liver). Libraries were barcoded and sequenced on an Illumina NextSeq instrument, in multiple 150 or 75 bp paired-end runs (along with other samples of amphibians and reptiles not used for this study) each of which combined 10–14 samples per High-Output NextSeq kit. To assemble the transcriptomes, reads were quality trimmed and filtered using fastp (Chen et al. 2018) and de novo assembled using Trinity v. 2.1.0 (Grabherr et al. 2011) following published protocols (Haas et al. 2013). The expression of BovB was assessed by BLASTN searches (Altschul et al. 1990) with BovB_VA as the query.

### BovB Search in Amphibian Genomes

The total DNA of three Malagasy frogs (*B. goudotii*, *Mt. betsileo*, and *Md. betsileanus*) was extracted from fresh livers, as described above. Library construction and sequencing by GS-FLX (Roche, Basel, Switzerland) were outsourced to Eurofins Genomics K.K. (Tokyo, Japan), Hokkaido System Science Co. Ltd. (Hokkaido, Japan), and Takara Bio Inc. (Shiga, Japan). Redundant reads and short reads less than 50 bp were eliminated using CDHit 454 (Li and Godzik 2006) and Solexa QA (Cox et al. 2010), respectively. The BovB sequences were searched using RepeatMasker (Smit et al. 2013–2015), and the percentage of BovB in each genome was calculated.

The genome assemblies of 21 amphibian species available from NCBI as of 2021 were searched using Megablast with BovB_VA as the query. For the six species in which hits were found (*Bufo gargarizans*, *Leptobrachium leishanense*, *Pyxicephalus adspersus*, *Lithobates catesbeianus*, *Rana temporaria*, and *Geotrypetes seraphini*), we then conducted a RepeatMasker search with the same query. The percentage of BovB in the genome of each species was estimated.

### Fluorescence In situ Hybridization

The cells of snakes and frogs were obtained from tissues of each individual and fixed in Carnoy fixative (methanol:acetic acids = 3:1 mixture). The slide spreads were prepared according to the standard procedure and FISH was carried out as described previously (Tanabe et al. 1996, 2021). The BovB_VA sequence cloned in pUC57 (total 5,879 bp) were labeled by nick translation with digoxigenin-11-dUTP (Roche 11093088910) and used as probes. Since BovB_VA could not hybridize with bufonid BovBs due to low sequence similarity (<80%), we made a specific probe for bufonids. Specifically, BovB amplicon from *B. japonicus* was cloned in pCR2.1-TOPO (total ca. 6,700 bp) and used as the probe. Labeled DNA probes were hybridized for 24–36 h onto slide spreads. After hybridization, the slides were washed and detected with mouse antidigoxigenin antibody (Sigma D-8156) in the first layer and successively detected with sheep antimouse, Cy3-conjugated antibody (Jackson ImmunoResearch Laboratories, Inc., West Grove, PA) in the second layer. The slides were counterstained with DAPI and mounted in Vectashield Antifade (Vector Laboratories, Inc., Burlingame, CA). FISH images were captured and analyzed using a Leica DM5000B fluorescent microscope equipped with a CCD camera and CW4000 image analysis software (Leica Microsystems, Wetzlar, Germany).

### Dot Blot Hybridization

For each frog sample used in the dot blot analysis, 500 and 50 ng of total DNA were denatured in a solution containing 0.8 N NaOH at 95 °C and deposited onto a Biodyne B Nylon Membrane (Nihon Pall Ltd., Tokyo, Japan) using a FLE348AA dot blotter (Advantec MFS Inc., Tokyo, Japan). Around 250 and 25 ng of DNA were used in the specimens for which enough DNA was not available (supplementary data 2, Supplementary Material online). The membrane was rinsed in 2× standard saline citrate, air dried, and baked at 80 °C. To enhance our ability to detect BovB elements, we used two probes for hybridization. The first fragment was 526 bp long and corresponded to the 5′ side coding AP endonuclease, amplified in BovB_VA sequence cloned in pUC57 using the primers ME1_Fmod and BovB_VA_1201_Rev. The second fragment was 502 bp long and corresponded to the 3′ side coding reverse transcriptase, amplified using BovB_VA_1942_Fow and ME2_Rmod (supplementary table S5, Supplementary Material online). Both PCR products were gel-purified as described above and [a-32P]dCTP-labeled using BcaBEST Labeling Kits (Takara Bio). The membrane was first hybridized with the 5′ side probe using PerfectHyb Hybridization Solution (Toyobo Co. Ltd., Osaka, Japan), washed and exposed on imaging plate, and scanned using Typhoon 9500 (GE Healthcare, Chicago, IL). It was then stripped and hybridized with the 3′ side probe with the same method.

To approximate the number of BovB copies present in the frog genomes, eight dilutions (2.5, 10, 50, 75, 100, 250, 500, and 1,000 pg) of cloned BovB_VA were blotted onto a membrane. The number of two-probe sequences contained in each dilution was calculated using the formula “copy number = (amount in ng × number/mole)/(length in bp × ng/g × g/mole of bp),” as described and implemented on the website http://cels.uri.edu/gsc/cndna.html. Based on the copy number of each dilution calculated from the integrated density of the signal, we created a standard curve and estimated the total copy number for each species. To calculate the copy number per haploid genome, we used *C* values taken from the Animal Genome Database (Gregory 2019), and the haploid genome sizes were calculated using the formula (Dolezel et al. 2003) “haploid genome in bp = *C* value × 0.978 × 10^9^”. When multiple *C* values were given for one species, we used the average of these values. When a *C* value was not available for a species, we used the average within the most closely related taxa, as shown in Pyron and Wiens (2011).

## Supplementary Material

msac052_Supplementary_Data

## Data Availability

Sequence data that support the findings of this study (raw reads of BovB sequence, genome, and transcriptome) have been deposited in the Sequence Read Archive (SRA) with the accession code (Bioproject) PRJDB11263. All other data are available in the main text and the Supplementary material online.
