# Supplementary material for: Geography-Dependent Horizontal Gene Transfer from Vertebrate Predators to Their Prey
Source: Mol Biol Evol. 2022 Apr 11;39(4):msac052. doi: 10.1093/molbev/msac052 (PMC9007160; doi:10.1093/molbev/msac052)
Supplement: msac052_Supplementary_Data [file msac052_supplementary_data.zip › SupplementaryMaterial.pdf]

Supplementary Material for

Geography-dependent horizontal gene transfer from vertebrate predators to their prey

Chiaki Kambayashi, Ryosuke Kakehashi, Yusuke Sato, Hideaki Mizuno, Hideyuki Tanabe, Andolalao Rakotoarison, Sven Künzel, Nobuaki Furuno, Kazuhiko Ohshima, Yoshinori Kumazawa, Zoltán T. Nagy, Akira Mori, Allen Allison, Stephen C. Donnellan, Hidetoshi Ota, Masaki Hoso, Tetsuya Yanagida, Hiroshi Sato, Miguel Vences, Atsushi Kurabayashi

This file includes:

Supplementary Note

Supplementary Figures 1–9

Supplementary Tables 1–7

Supplementary References

Supplementary Data 1 and 2 are provided in a separate Excel file.

Supplementary Note

Detailed discussion of BovB HT

Adding to the snake-to-snake HTs shown in the main text (specifically typhlopoids and Malagasy boas), we identified other HT instances among snake lineages. The BovBs of the families Colubridae and Viperidae were derived within the clade of the family Elapidae, unlike the phylogeny based on orthologous genes (nodes #26 and 31; see supplementary fig. 9 and table 6, Supplementary Material online). Moreover, several snake BovBs were derived from the clade of different families (nodes #27, 29, 33, 34, and 35). The BovB sequences of these families reflect little of the general phylogenetic views and show low nucleotide divergences, suggesting that HT among snakes may occur more frequently than previously thought (Walsh et al. 2013; Puinongpo et al. 2020).

In the clade of the snake family Pseudoxyrhophiidae, BovBs of the microhylid frogs belonging to Cophylinae (*Cophyla* and *Plethodontohyla*) form a monophyletic group with that of a mantellid frog, *Gephyromantis boulengeri* (node #45). Similarly, the BovB of a ptychadenid frog, *Ptychadena mascareniensis*, appears in the clade of the *Boophis* frogs of the family Mantellidae (node #54). These findings of closely related BovBs among phylogenetically distant frogs suggest that BovB HT has also occurred between frogs.

The BovBs of four frog families (Dicroglossidae, Mantellidae, Microhylidae, and Ranidae) are derived from the clade of Henophidia (primitive snakes). When we adopt a parsimonious criterion on the HT numbers and consider the branching ages of each taxonomic group (Kumar et al. 2017), the following HT events could be suggested. First the BovB was transferred from the ancestor of Henophidia (Boidae, Loxocemidae, and Pythonidae) to that of the microhylid subfamilies, Asterophryinae and Dyscophinae (node #8). Then BovB HTs have occurred from the ancestor of Dyscophinae to that of Ranidae (*Babina*, *Odorrana*, and *Pelophylax*) (node #9) and a mantellid, *Aglyptodactylus madagascariensis* (node #10), and from the ancestors of Asterophryinae to that of Dicroglossidae (*Euphlyctis*, *Fejrvarya*, and *Hoplobatrachus*) (node #12) and a ranid, *Papurana* sp. (node #13). In this clade, the BovB of the xenopeltid snake, *Xenopeltis unicolor*, which is distributed in Southeast Asia and known to derive from the ancestor of Loxocemidae and Pythonidae, is closely related to that of Asterophryinae. Although asterophryine taxa are widely distributed in Oceania, the most primitive asterophryine, *Gastrophrynoides*, is distributed in Southeast Asia and is considered that the asterophryine ancestor migrated from Southeast Asia to Oceania 25–48 Ma (Kurabayashi et al. 2011). It is therefore possible that horizontal transfer of BovB occurred there

from the ancestor of Asterophryinae to that of *Xenopeltis unicolor* from frogs to snakes (node #11).

Several BovBs of Sauria also emerged in the snake lineages. The BovBs of the Oceanian lizards, including Agamidae and Scincidae, were derived from the common ancestor of Henophidia (node #3). The BovBs of a gecko genus, *Gehyra*, and a chameleon, Chamaeleonidae sp., were closely related to that of a snake, *Pareas iwasakii* of the family Pareidae (node #19). The BovB of a Malagasy gecko, *Phelsuma lineata*, was derived from the clade of the Malagasy snake family Pseudoxyrhophiidae (node #48). These cases indicate that the newly integrated BovBs from snakes are more dominant in these lizard lineages than the BovBs that originated in the common ancestor of squamates.

Some of the parasites used in this study had different BovBs among closely related species and within species. The BovBs of three Malagasy chiggers belonging to the family Trombiculidae were derived from the clades of *Boa* snakes (node #16), the chameleon family Chamaeleonidae (node #20) and the snake family Pseudoxyrhophiidae (node #51). Chiggers only suck blood once, as larvae (Mullen and Oconnor 2002), so contamination of DNA from sources other than their hosts is unlikely. We also found that two specimens of the Malagasy nematode, identified based on 18S rRNA sequences as belonging to the same species (*Raillietnema* sp.), have different BovBs, derived from the clade of the hyperoliid frog genus *Heterixalus* (node #42) and the mantellid frog genus *Mantidactylus* (node #52). Since the BovB sequences detected from these chiggers and nematodes differed from those of their hosts (sequence similarity < 98%), the possibility of contamination is low. This evidence indicates that closely related parasites can transport a variety of BovBs to their hosts through frequent HT incidents or carriage of bacteria and viruses that have been considered to be candidate vectors of HTs among some eukaryotes (Liu et al. 2010; Gilbert et al. 2014; Ortiz et al. 2015).

The origin of the parasite BovBs, which was reported in a previous study, was also clarified in more detail. The tick *Amblyomma limbatum*, collected from an Oceanian lizard, is known to have BovBs similar to that of the elapid and viperid snakes (Walsh et al. 2013), but this study showed that the BovB was derived from the primitive lineage of Caenophidia (node #23) and was most closely related with that of an Oceanian frog, *Cornufer pelewensis* of the family Ceratobatrachidae (node #24). The BovB of a bedbug, *Cimex lectularius*, has 80% homology with those of the elapid, colubrid, and viperid snakes (Ivancevic et al. 2018; Puinongpo et al. 2020). However, our study revealed that its BovB is most closely related to that of *Mimophis* cf. *mahfalensis* belonging to the snake family Psammophiidae, with 94% homology (node #37).

BovB detection in amphibian genomes

We conducted a Megablast search for BovBs in the genome assemblies of 21 amphibian species available from the NCBI and obtained hits from five frogs and one caecilian. We detected BovBs in the frogs that belong to the same family as *Bufo gargarizans* (Bufonidae), *Leptobrachium leishanense* (Megophryidae), *Lithobates catesbeianus*, and *Rana temporaria* (Ranidae). This result confirms that the BovB detected in frogs in this study was not due to DNA contamination. For *Pyxicephalus adspersus* (Pyxicephalidae) and *Geotrypetes seraphini* (Dermophiidae), the longest BovB sequences obtained in the RepeatMasker search (Smit et al. 2013–2015) were compared with the BovB sequences used in this study, which were most closely related to the BovB of a boid snake, *Eryx colubrinus* (72%), and a hyrax, *Procavia capensis* (61%). No hits were found in the Megablast search from *Xenopus laevis* and *X. tropicalis*, in which the presence of BovB was previously shown (Ivancevic et al. 2018). Our phylogenetic analysis revealed that these two BovB sequences are more primitive than the BovB of an anole lizard, *Anolis carolinensis* (supplementary fig. 5, Supplementary Material online), indicating that they are different from the squamate-type BovBs focused on in this study.

a

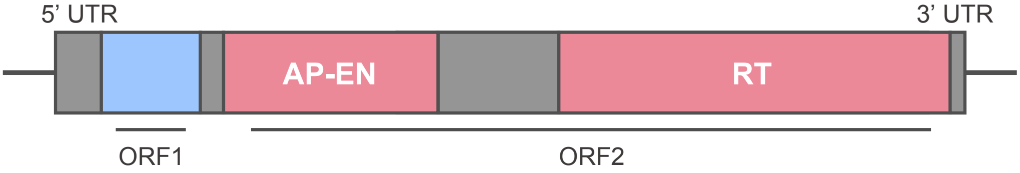

b

```
       MTLEDEPLRSEGIQYATGEEQRATTSSPRKNEATGPKPKGHSAVDVSGGERKVRCCKDF
AP-EN  FSIGTWNVRSMNQGKLDVVKQEMTRLNIDILGVSELKWTGMGEFNSDDHQVYYCGQESL
       RRNGVAFTVNKRVEKAILGYNPQNDRMISVRIQGKPFNITVVQVYAPTTSAEEDEIDRFCE
       ALQHLIELTPKNDVLIIMGDWNAKVGSQKITRITGKFGLGVQNEAGHRLIEFCQENTMVIAN
       TLFQQPKRRLYTWTSPDGQYRNQIDYVLCSQRWRSSIQSVKTRPGADCGSDHELLVAKF
       RLKLKKVGKSTRPLRYELNHIPVEYTVEVTNRFKELDLIDRVPEELWTEVRNIV*EVATKTIP
       KKKKCKKAKWLSEEALQIADERREAKGKGEKEIYAQLNAEFQTIARRDKNAFLNEQCKEIE
       ENNRIGRTRDPFKKIGEMKGTFHAKMGMIKDQNGRDLTEAEEIKKRWQNYTEELYKNELN
       VPDNLNEVVTDLEPDILECEVKWALEKLSNNKASGGGNIPAELFKILKDNAVKVLHSICQQI
RT     WKTQQWPQDWKRSVYIPIPKRGSAKECSNYRTIALISHASKVMLKILQARLQQYVDRELP
       EVQAGFRRGRGTRDQIANIRWLMEKAREFQKNIYFCFIDYAKAFDCVDHNKLWQVLKEM
       GVPDHLICLLRNLYAGQEATVRTGHGTTDWFKIGKGVRQGCILLPCLFNLYAEHIMRKAGL
       DESKVGIKIAGRNINNLRYADDTTLMAESEEELKSLLLRVKKESAKLGLKLNIKKTKIMASNP
       LNSWQIDGEEMEVVTDFIFLGSKITADGDCSQEIKRRTLLRRKAMANLDSTLKSRDITLSTK
       VRIVKAMVFPVAMYGSESWTIKKAERQRIEAFELWCWRRLLRVPWTARRSNRSVLEEINP
       DCSLEGQILKLKLKYFGHLMRRKDSLEKSLMLGKIEGNKRMGRQRMRVLDGVTEAVGVS
       LNGLQKMVEDRKAWRNIVHRVAMGRTRLRS
```

Supplementary Fig. 1. Structure and amino acid sequence of BovB_VA. a, The structure of BovB_VA, the full-length consensus sequence of BovB in the nose-horned viper (*Vipera ammodytes*). AP-EN, AP endonuclease; RT, reverse transcriptase; UTR, untranslated region; ORF, open reading frame. b, The amino acid sequence of ORF2. The locations of the probes used for dot blot analysis are highlighted in orange. Modified from Zupunski et al. (2001).

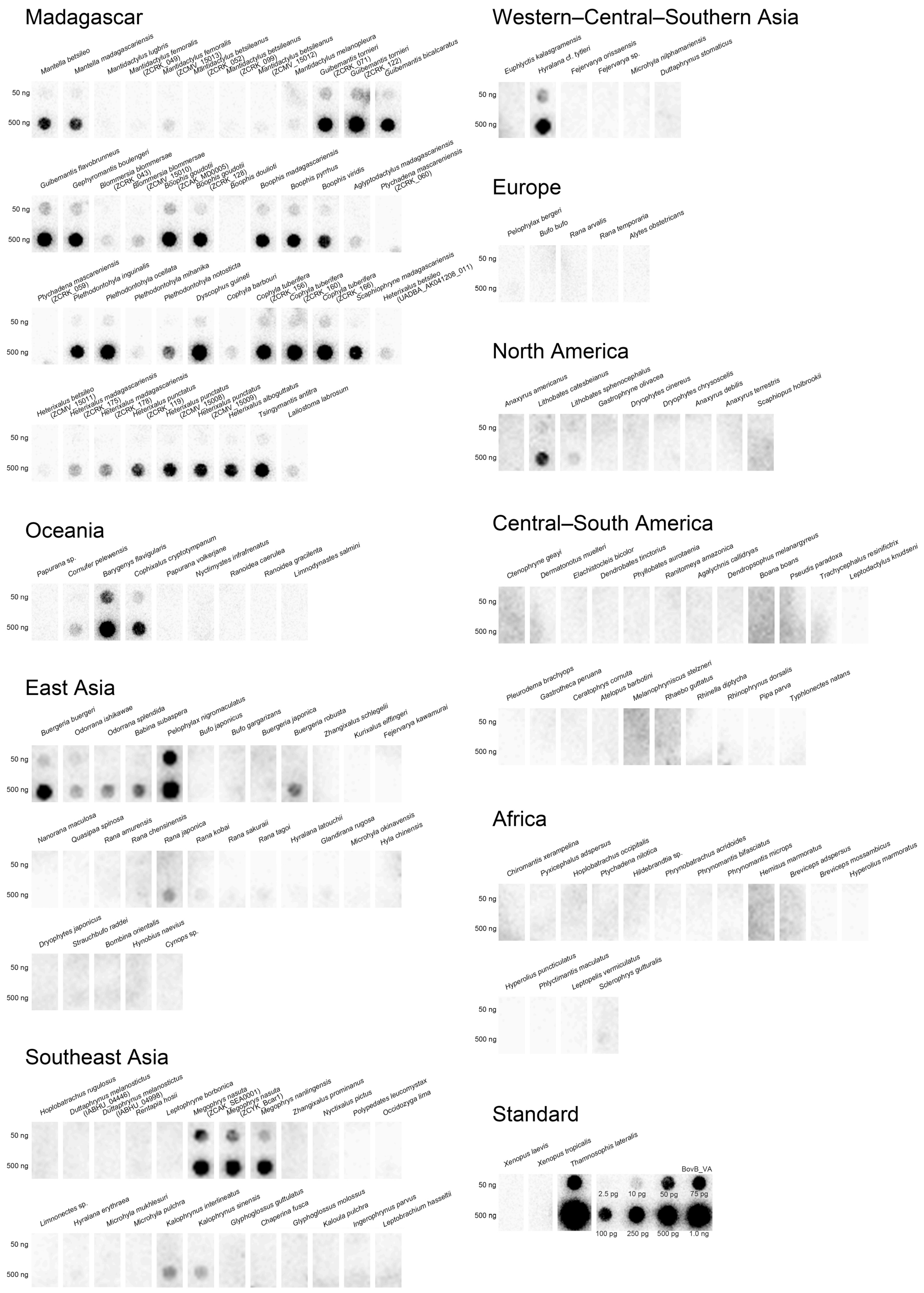

Supplementary Fig. 2. Dot blot hybridization with AP endonuclease sequence probe. BovB was detected in frog genomes by dot blot analysis using the 5' side partial sequences of BovB_VA corresponding to AP endonuclease region (526 bp) as a probe.

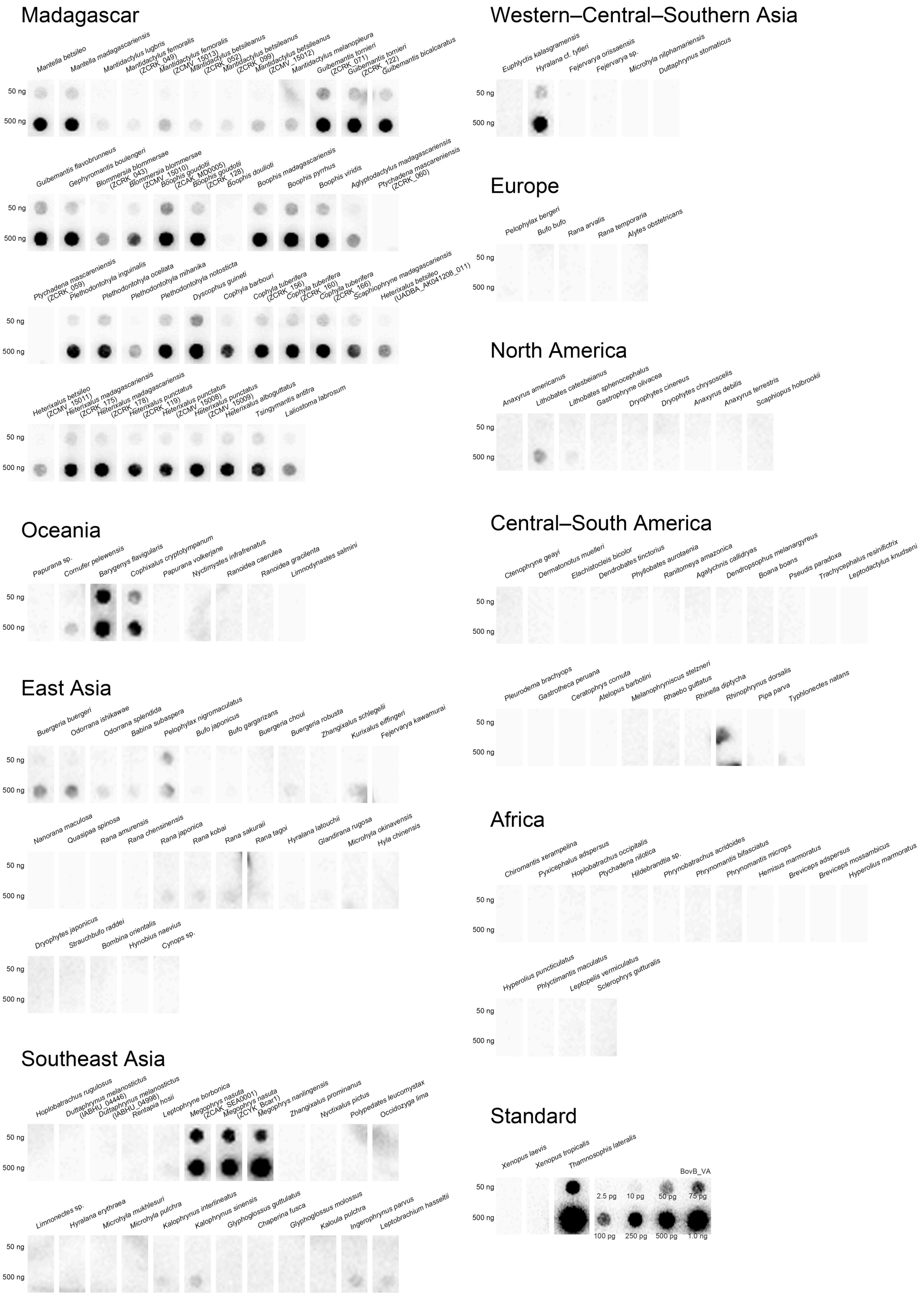

Supplementary Fig. 3. Dot blot hybridization with reverse transcriptase sequence probe. BovB was detected in frog genomes using the 3’ side partial sequences of BovB_VA corresponding to reverse transcriptase region (502 bp) as a probe.

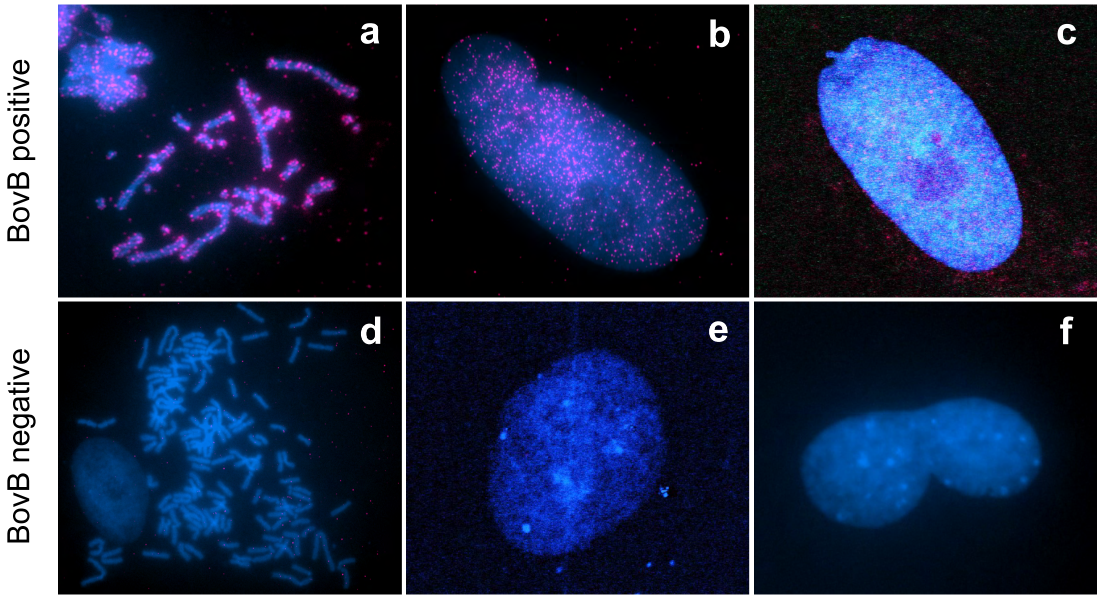

Supplementary Fig. 4. The FISH visualization of BovBs. BovB sequences were detected on the chromosomes or nuclei of snake and frog species. The nuclei and chromosomes were counterstained with DAPI. a, *Elaphe quadrivirgata*; b, *Mantella betsileo*; c, *Bufo japonicus*; d, *Xenopus laevis*; e, *Xenopus tropicalis*; f, *Buergeria japonica*. BovB was detected using full BovB_VA sequence cloned in *E. coli* pUC57 vector (a, b, d–f) and the BovB amplicon of *B. japonicus* cloned in pCR2.1-TOPO vector (c) as probes.

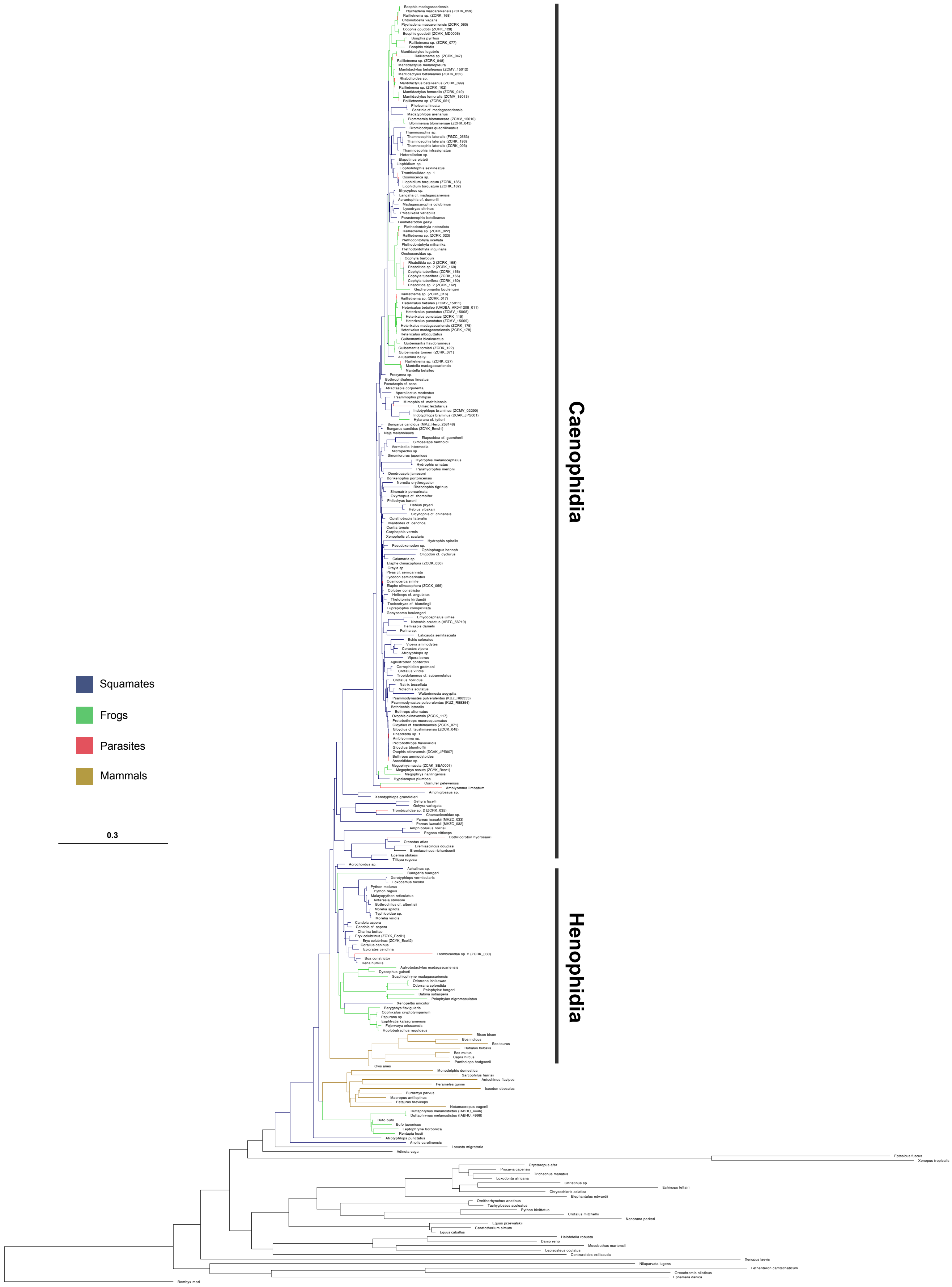

Supplementary Fig. 5. ML tree of 285 BovB sequences. The ML phylogeny reconstructed from the 211 BovB sequences obtained in this study plus 74 known sequences.

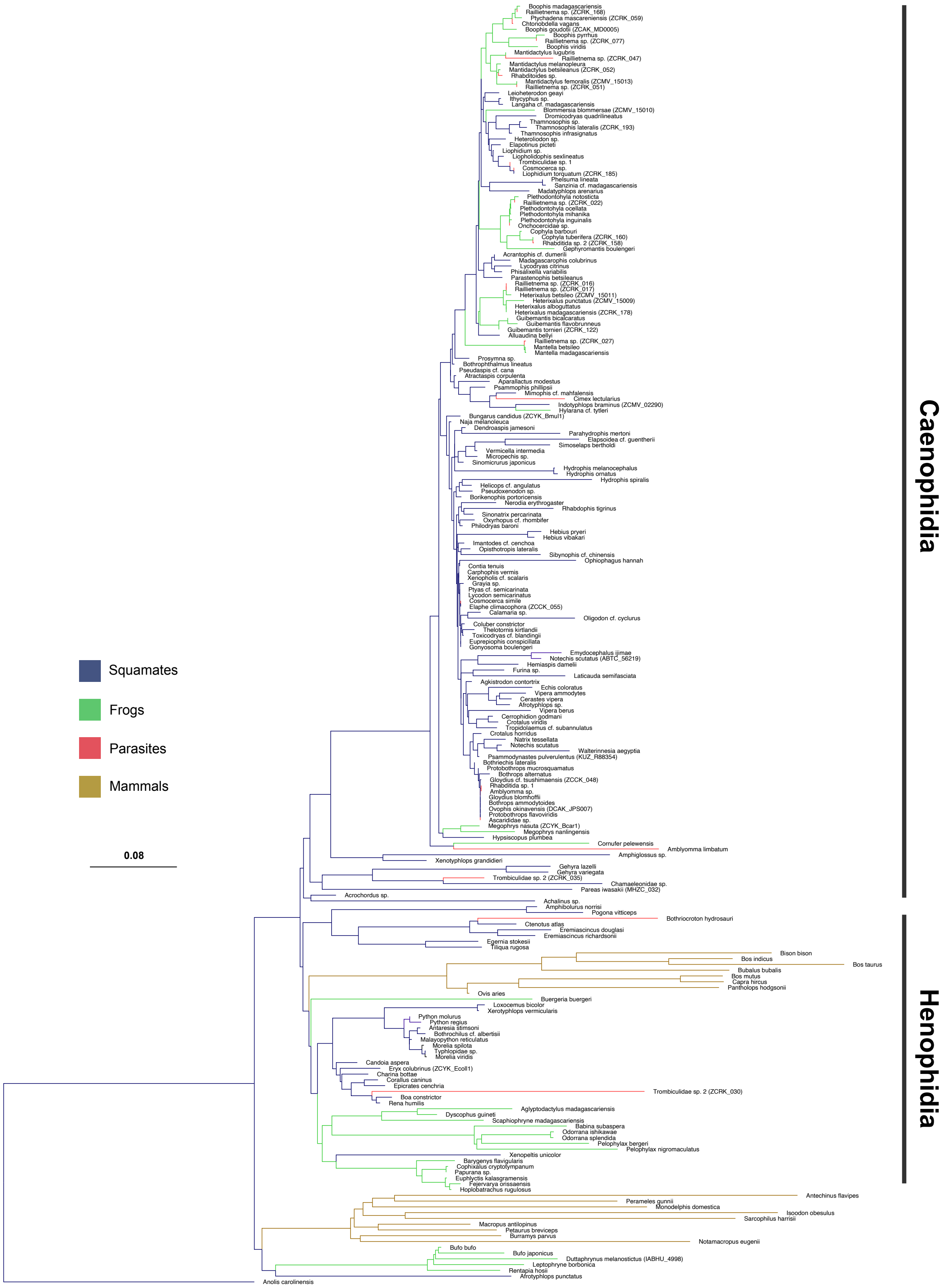

Supplementary Fig. 6. ML analysis results of 222-OTUs BovB sequences. The ML phylogeny of the 222 BovB sequences reconstructed by RAxML program (see the main text) is shown.

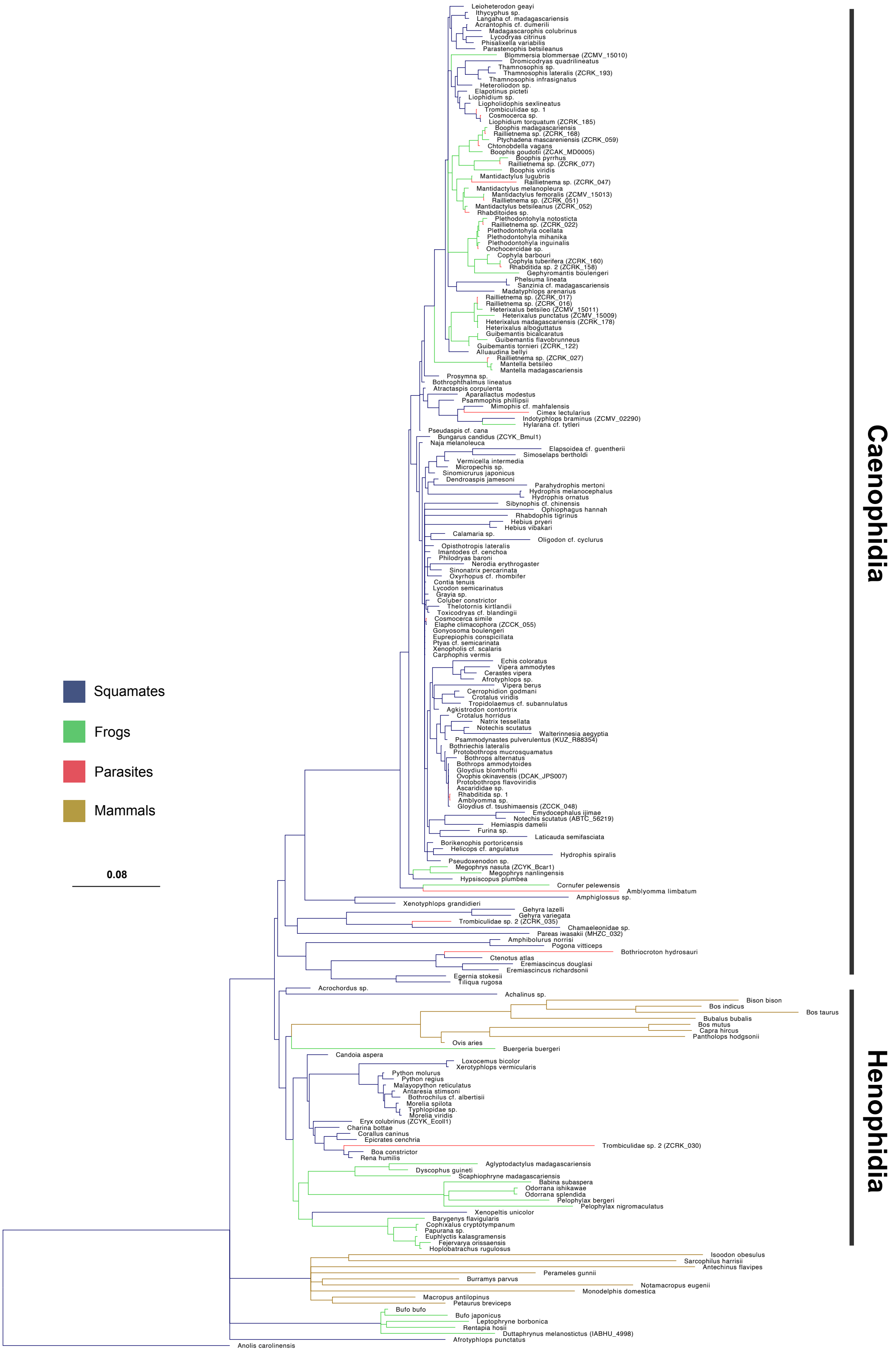

Supplementary Fig. 7. BI analysis result of 222-OTUs BovB sequences. The BI phylogeny estimated using the BovB sequences inferred by MrBayes software (see the main text) is shown.

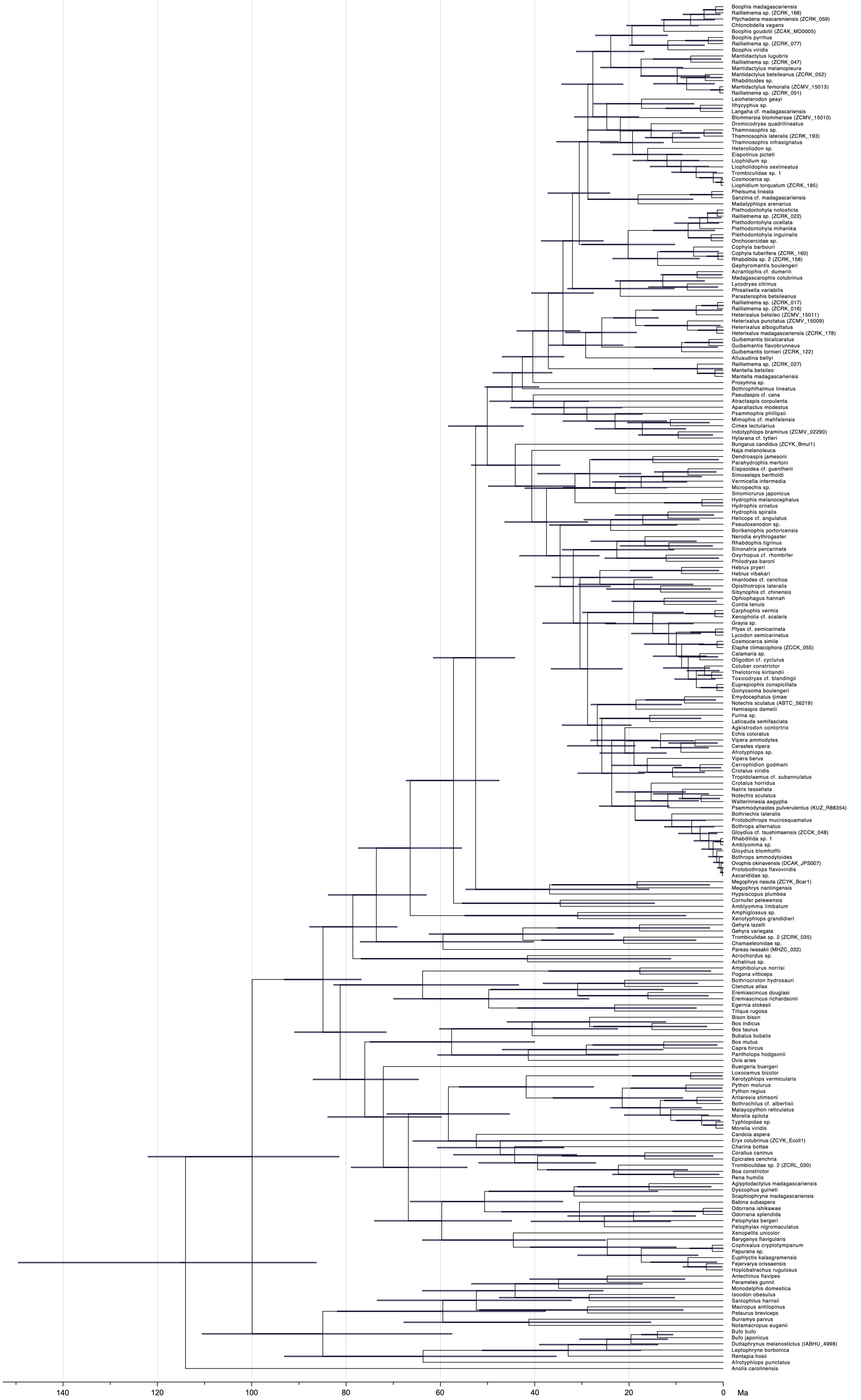

Supplementary Fig. 8. Time tree of squamate-type BovBs. The time tree with mean node age inferred based on the 222-OTUs ML topology by BEAST software (see the main text) is shown. The 95% confidence intervals of the divergence ages were depicted as blue bars at each node.

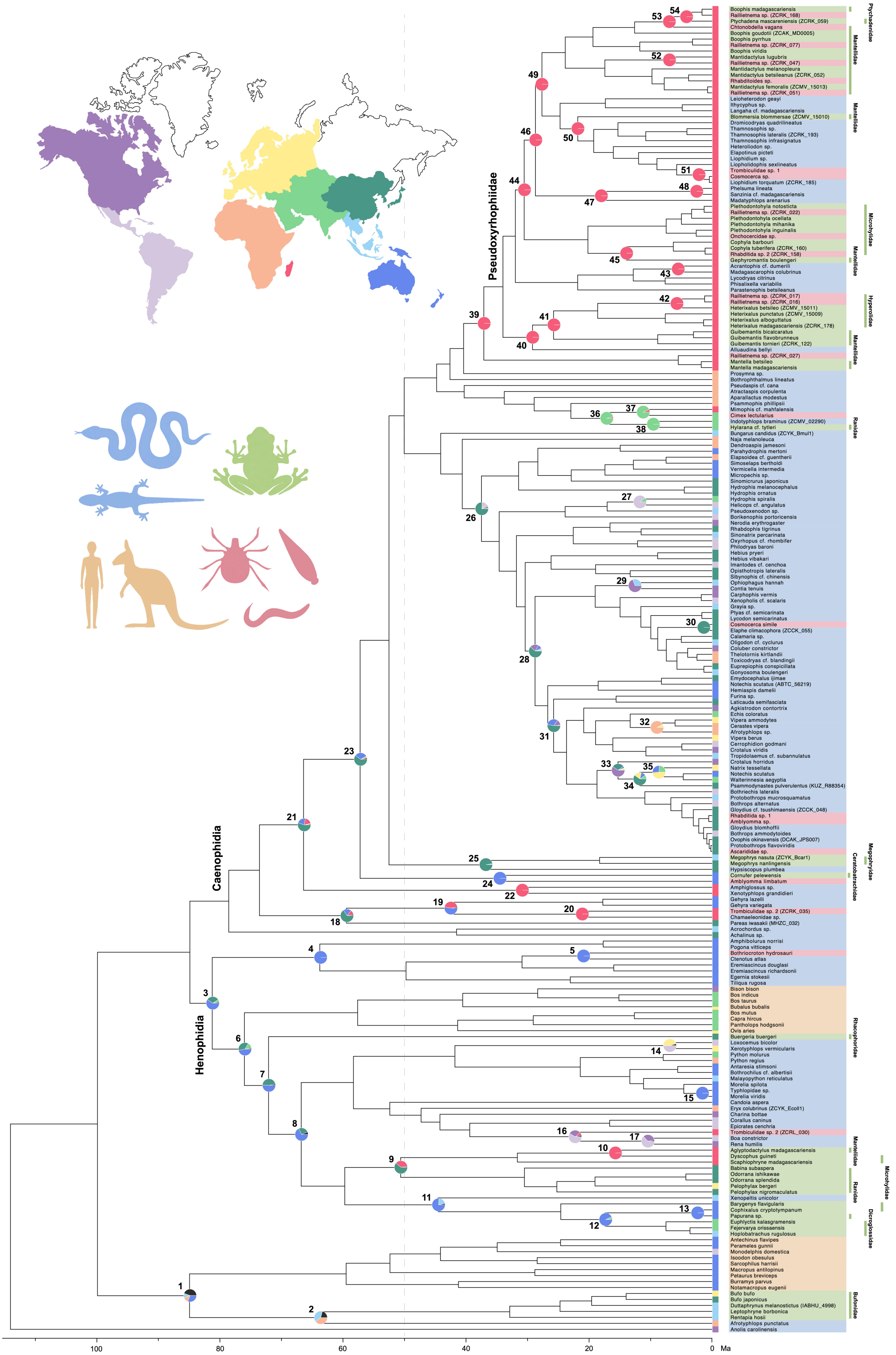

Pseudoxyrhophiidae
Caenophidia
Henophidia
Boophis madagascariensis
Raillietnema sp. (ZCRK_168)
Ptychadena mascareniensis (ZCRK_059)
Chtonobdella vagans
Boophis goudotii (ZCAK_MD0005)
Boophis pyrrhus
Raillietnema sp. (ZCRK_077)
Boophis viridis
Mantidactylus lugubris
Raillietnema sp. (ZCRK_047)
Mantidactylus melanopleura
Mantidactylus betsileanus (ZCRK_052)
Rhabditoides sp.
Mantidactylus femoralis (ZCMV_15013)
Raillietnema sp. (ZCRK_051)
Leioheterodon geayi
Ithycyphus sp.
Langaha cf. madagascariensis
Blommersia blommersae (ZCMV_15010)
Dromicodryas quadrilineatus
Thamnosophis sp.
Thamnosophis lateralis (ZCRK_193)
Thamnosophis infrasignatus
Heteroliodon sp.
Elapotinus picteti
Liophidium sp.
Liopholidophis sexlineatus
Trombiculidae sp. 1
Cosmocerca sp.
Liophidium torquatum (ZCRK_185)
Phelsuma lineata
Sanzinia cf. madagascariensis
Madatyphlops arenarius
Plethodontohyla notosticta
Raillietnema sp. (ZCRK_022)
Plethodontohyla ocellata
Plethodontohyla mihanika
Plethodontohyla inguinalis
Onchocercidae sp.
Cophyla barbouri
Cophyla tuberifera (ZCRK_160)
Rhabditida sp. 2 (ZCRK_158)
Gephyromantis boulengeri
Acrantophis cf. dumerili
Madagascarophis colubrinus
Lycodryas citrinus
Phisalixella variabilis
Parastenophis betsileanus
Raillietnema sp. (ZCRK_017)
Raillietnema sp. (ZCRK_016)
Heterixalus betsileo (ZCMV_15011)
Heterixalus punctatus (ZCMV_15009)
Heterixalus alboguttatus
Heterixalus madagascariensis (ZCRK_178)
Guibemantis bicalcaratus
Guibemantis flavobrunneus
Guibemantis tornieri (ZCRK_122)
Alluaudina bellyi
Raillietnema sp. (ZCRK_027)
Mantella betsileo
Mantella madagascariensis
Prosymna sp.
Bothrophthalmus lineatus
Pseudaspis cf. cana
Atractaspis corpulenta
Aparallactus modestus
Psammophis phillipsii
Mimophis cf. mahfalensis
Cimex lectularius
Indotyphlops braminus (ZCMV_02290)
Hylarana cf. tytleri
Bungarus candidus (ZCYK_Bmul1)
Naja melanoleuca
Dendroaspis jamesoni
Parahydrophis mertoni
Elapsoidea cf. guentherii
Simoselaps bertholdi
Vermicella intermedia
Micropechis sp.
Sinomicrurus japonicus
Hydrophis melanocephalus
Hydrophis ornatus
Hydrophis spiralis
Helicops cf. angulatus
Pseudoxenodon sp.
Borikenophis portoricensis
Nerodia erythrogaster
Rhabdophis tigrinus
Sinonatrix percarinata
Oxyrhopus cf. rhombifer
Philodryas baroni
Hebius pryeri
Hebius vibakari
Imantodes cf. cenchoa
Opisthotropis lateralis
Sibynophis cf. chinensis
Ophiophagus hannah
Contia tenuis
Carphophis vermis
Xenopholis cf. scalaris
Grayia sp.
Ptyas cf. semicarinata
Lycodon semicarinatus
Cosmocerca simile
Elaphe climacophora (ZCCK_055)
Calamaria sp.
Oligodon cf. cyclurus
Coluber constrictor
Thelotornis kirtlandii
Toxicodryas cf. blandingii
Euprepiophis conspicillata
Gonyosoma boulengeri
Emydocephalus ijimae
Notechis scutatus (ABTC_56219)
Hemiaspis damelii
Furina sp.
Laticauda semifasciata
Agkistrodon contortrix
Echis coloratus
Vipera ammodytes
Cerastes vipera
Afrotyphlops sp.
Vipera berus
Cerrophidion godmani
Crotalus viridis
Tropidolaemus cf. subannulatus
Crotalus horridus
Natrix tessellata
Notechis scutatus
Walterinnesia aegyptia
Psammodynastes pulverulentus (KUZ_R88354)
Bothriechis lateralis
Protobothrops mucrosquamatus
Bothrops alternatus
Gloydius cf. tsushimaensis (ZCCK_048)
Rhabditida sp. 1
Amblyomma sp.
Gloydius blomhoffii
Bothrops ammodytoides
Ovophis okinavensis (DCAK_JPS007)
Protobothrops flavoviridis
Ascarididae sp.
Megophrys nasuta (ZCYK_Bcar1)
Megophrys nanlingensis
Hypsiscopus plumbea
Cornufer pelewensis
Amblyomma limbatum
Amphiglossus sp.
Xenotyphlops grandidieri
Gehyra lazelli
Gehyra variegata
Trombiculidae sp. 2 (ZCRK_035)
Chamaeleonidae sp.
Pareas iwasakii (MHZC_032)
Acrochordus sp.
Achalinus sp.
Amphibolurus norrisi
Pogona vitticeps
Bothriocroton hydrosauri
Ctenotus atlas
Eremiascincus douglasi
Eremiascincus richardsonii
Egernia stokesii
Tiliqua rugosa
Bison bison
Bos indicus
Bos taurus
Bubalus bubalis
Bos mutus
Capra hircus
Pantholops hodgsonii
Ovis aries
Buergeria buergeri
Loxocemus bicolor
Xerotyphlops vermicularis
Python molurus
Python regius
Antaresia stimsoni
Bothrochilus cf. albertisii
Malayopython reticulatus
Morelia spilota
Typhlopidae sp.
Morelia viridis
Candoia aspera
Eryx colubrinus (ZCYK_Ecoll1)
Charina bottae
Corallus caninus
Epicrates cenchria
Trombiculidae sp. 2 (ZCRL_030)
Boa constrictor
Rena humilis
Aglyptodactylus madagascariensis
Dyscophus guineti
Scaphiophryne madagascariensis
Babina subaspera
Odorrana ishikawae
Odorrana splendida
Pelophylax bergeri
Pelophylax nigromaculatus
Xenopeltis unicolor
Barygenys flavigularis
Cophixalus cryptotympanum
Papurana sp.
Euphlyctis kalasgramensis
Fejervarya orissaensis
Hoplobatrachus rugulosus
Antechinus flavipes
Perameles gunnii
Monodelphis domestica
Isoodon obesulus
Sarcophilus harrisii
Macropus antilopinus
Petaurus breviceps
Burramys parvus
Notamacropus eugenii
Bufo bufo
Bufo japonicus
Duttaphrynus melanostictus (IABHU_4998)
Leptophryne borbonica
Rentapia hosii
Afrotyphlops punctatus
Anolis carolinensis
Ptychadenidae
Mantellidae
Mantellidae
Microhylidae
Mantellidae
Hyperolidae
Mantellidae
Ranidae
Megophryidae
Ceratobatrachidae
Rhacophoridae
Mantellidae
Ranidae
Microhylidae
Dicroglossidae
Bufonidae
100
80
60
40
20
0
Ma

Supplementary Fig. 9. Estimation of ancestral areas of HTs. The areas where the BovB HTs had occurred were estimated based on the 222-OTUs chronogram using the DIVALIKE+J model implemented in BioGeoBEARS software (see the main text). The pie charts at each node represent the set of possible ancestral areas, and the colour is associated with the area on the map. The node numbers correspond to supplementary table 6, Supplementary Material online.

Supplementary Table 1. The percentage of amphibian genome sequence contributed by BovB.

Family	Species	BovB base count	Genome base count	BovB coverage	Accession number
Mantellidae	*Boophis goudotii*	249,927	47,278,508	0.53%	PRJDB11263
Mantellidae	*Mantella betsileo*	148,950	111,622,769	0.13%	PRJDB11263
Mantellidae	*Mantidactylus betsileanus*	30,072	28,523,085	0.11%	PRJDB11263
Bufonidae	*Bufo gargarizans*	237,696	4,545,465,442	0.0052%	GCA_014858855.1
Megophryidae	*Leptobrachium leishanense*	358,785	3,549,060,464	0.01%	GCA_009667805.1
Pyxicephalidae	*Pyxicephalus adspersus*	81,463	1,563,367,516	0.0052%	GCA_004786255.1
Ranidae	*Lithobates catesbeianus*	656,769	6,250,353,185	0.011%	GCA_002284835.2
Ranidae	*Rana temporaria*	297,767	4,282,724,626	0.007%	GCA_009802015.1
Dermophiidae	*Geotrypetes seraphini*	2,649,618	3,779,430,019	0.07%	GCA_902459505.1
Bufonidae	*Bufo bufo*	No blast hit	5,044,762,059	–	GCA_905171765.1
Bufonidae	*Rhinella marina*	No blast hit	2,551,760,146	–	GCA_900303285.1
Dendrobatidae	*Oophaga pumilio*	No blast hit	3,493,380,730	–	GCA_009801035.1
Dicroglossidae	*Nanorana parkeri*	No blast hit	2,053,867,363	–	GCA_000935625.1
Limnodynastidae	*Limnodynastes dumerilii*	No blast hit	2,378,679,715	–	GCA_011038615.1
Limnodynastidae	*Platyplectrum ornatum*	No blast hit	1,065,311,793	–	GCA_016617825.1
Pipidae	*Xenopus laevis*	No blast hit	2,718,433,805	–	GCA_001663975.1
Pipidae	*Xenopus tropicalis*	No blast hit	1,451,301,209	–	GCA_000004195.4
Scaphiopodidae	*Scaphiopus couchii*	No blast hit	484,148,731	–	GCA_009364435.1
Scaphiopodidae	*Scaphiopus holbrookii*	No blast hit	710,849,620	–	GCA_009364455.1
Scaphiopodidae	*Spea bombifrons*	No blast hit	765,304,928	–	GCA_009364475.1
Scaphiopodidae	*Spea multiplicata*	No blast hit	1,075,972,709	–	GCA_009364415.1
Ambystomatidae	*Ambystoma mexicanum*	No blast hit	32,396,370,977	–	GCA_002915635.2
Rhinatrematidae	*Rhinatrema bivittatum*	No blast hit	5,319,239,201	–	GCA_901001135.1
Siphonopidae	*Microcaecilia unicolor*	No blast hit	4,685,939,420	–	GCA_901765095.1

Supplementary Table 2. The indices of distance between the topologies on the 222-OTUs ML tree and that on a tree constructed based on orthologous genes.

	I_{cong} (P-value)	Triples
squamates		
species	2.4 (2.5×10^{-15})	0.52
genera	2.2 (3.1×10^{-13})	0.53
families	1.7 (1.3×10^{-6})	0.63
frogs		
species	1.6 (1.1×10^{-5})	0.64
genera	1.4 (1.3×10^{-3})	0.64
families	1.4 (5.2×10^{-3})	0.80

The larger the I_{cong} index value and the smaller the triples value, the more congruent the trees are. The p-value smaller than 0.05 indicates that the trees are more congruent than expected by chance.

Supplementary Table 3. Frequency of horizontal transfer (HT) at both species and genus levels in each geographic region and comparisons with those in Madagascar.

Region	Number of HT occurrence	Number of BovB-positive species	p-value (vs. Madagascar)	Number of BovB-positive genera	p-value (vs. Madagascar)
Madagascar	14	57	–	37	–
Oceania	5	32	0.14	27	5.1×10^{-3}
East Asia	5	53	3.4×10^{-3}	41	3.1×10^{-4}
Central–South America	3	36	1.1×10^{-3}	35	6.3×10^{-6}
Western–Central–Southern Asia	2	12	0.11	11	8.9×10^{-3}
North America	2	20	4.8×10^{-3}	14	1.0×10^{-3}
Africa	1	36	2.8×10^{-9}	31	2.8×10^{-11}
Europe	1	9	0.013	7	1.0×10^{-3}
Southeast Asia	0	32	$< 1.8 \times 10^{-15}$	29	$< 1.8 \times 10^{-15}$

The numbers of reptile and amphibian taxa and the numbers of HT occurrences among them within the past 50 Ma were summarized. P-value was adjusted using holm correction.

Supplementary Table 4. Frequency of snake species known to consume frogs in each region and comparisons with those in Madagascar.

Region	Percentage	P-value (vs. Madagascar)
Madagascar	39% (11/28)	–
Oceania	45% (13/29)	1.00
East Asia	49% (36/74)	1.00
Central–South America	37% (163/435)	1.00
Western–Central–Southern Asia	24% (29/120)	1.00
North America	34% (39/115)	1.00
Africa	43% (55/129)	1.00
Europe	21% (4/19)	1.00
Southeast Asia	23% (18/77)	1.00
Average	36% (368/1026)	–

Raw numbers of species used to calculate percentages are in parentheses. P-value was adjusted using holm correction.

Supplementary Table 5. PCR primers used in this study.

Gene	Primer name	Primer sequence (5'→3')	Source
Cytb	L14910	GACCTGTGATMTGAAAACCAYCGTTGT	Burbrink et al. (2000)
	H16064	CTTTGGTTTACAAGAACAATGCTTTA	
	Cytb1	CCATCCAACATCTCAGCATGATGAAA	Kocher et al. (1989)
	Cytb2	CCCTCAGAATGATATTTGTCCTCA	
16S rRNA	16Sar	CGCCTGTTTATCAAAAACAT	Bossuyt and Milinkovitch (2000)
	16Sbr	CGGTCTGAACTCAGATCACG	
18S rRNA	18S_Fow_11_31	TACCTGGTTGATYCTGCCAGT	This study
	18S_Fow_92_109	GAAACYGCGAAYGGCTCA	
	18S_Rev_1252_1272	TTYCCCGTGTTGAGTCAAATT	
	18S_Rev_1692_1714	CGGTGTGTACAAAGGGCAGGGAC	
BovB	ME1_Fmod	ACAGTARTYCAAGYCTAYRCYCCAAC	This study
	BovB_ORF5_FowN	AGRTCGGAAGGTRTCCAATATGCTAC	
	BovB_230F	AGGTCRGAAGRYGYCCAWYATGCTAC	
	BovB_670F	AGTARTCCAAGTCTATGCMCCAAYCA	
	BovB_VA_1942_Fow	GCACTCATTTCTCATGCTAGTAAAGT	
	ME2_Rmod	CWGCAWATCTGAGGTTGTTRAKATTTCT	
	BovB_ORF3_RevN	GTGAYKCCATCCAGCCAYCTCATYCTCTG	
	BovB_2460R	GCCATYAGRGTGGTRTCRTCTGCATAYCT	
	BovB_2820R	TTAYRGTCCARCTYTCRCABCCATACAT	
	BovB_3090R	CYRTCYARCCAYCTCATYCTCTGYCG	
	BovB_3200R	AAGTCGTGTCCGACYCWTCGYRACCCYATG	
	BovB_VA_1201_Rev	ATCTATTTGTCACCTCTACT	

Supplementary Table 6. The nodes of BovB HTs in the ML and BI trees of 222-OTUs.

Node	Date (Ma)	Taxa involved in the HT	HT region	BPs	BPPs (Unresolved: <90)
1	84.9	*Afrotyphlops* or Bufonidae – Marsupialia	–	34	Unresolved (polytomy)
2	63.7	*Afrotyphlops punctatus* – Bufonidae	–	39	Unresolved (polytomy)
3	81.2	Henophidia – Common ancestor of Agamidae or Scincidae	–	12	–
4	63.8	Agamidae – Scincidae	–	91	100
5	20.9	*Ctenotus atlas* – *Bothriocroron hydrosauri*	Oceania	67	81
6	76.0	Henophidia – Bovidae	–	34	98
7	72.1	Henophidia – *Buergeria buergeri*	–	51	–
8	66.8	Henophidia – Microhylidae	–	39	98
9	50.6	Dyscophinae – Ranidae	–	48	99
10	15.8	*Dyscophus guineti* – *Aglyptodactylus madagascariensis*	Madagascar	100	100
11	44.5	Asterophryinae – *Xenopeltis unicolor*	Oceania	56	99
12	17.4	*Cophixalus cryptotympanum* – Dicroglossidae	Oceania	98	96
13	2.3	*Cophixalus cryptotympanum* – *Papurana* sp.	Oceania	100	100
14	6.9	*Loxocemus bicolor* – *Xerotyphlops vermicularis*	Central–South America	100	100
15	1.6	*Morelia viridis* – Typhlopidae sp.	Oceania	100	100
16	22.3	*Boa constrictor* – Trombiculidae sp. 2 (ZCRK_030)	Central–South America	100	100
17	10.5	*Boa constrictor* – *Rena humilis*	Central–South America	100	Unresolved
18	59.4	*Pareas iwasakii* – *Gehyra* or Chamaeleonidae or Trombiculidae	–	39	95
19	42.5	*Gehyra* – Chamaeleonidae or Trombiculidae	Oceania	100	100
20	21.1	Chamaeleonidae sp. – Trombiculidae sp. 2 (ZCRK_035)	Madagascar	100	100
21	66.4	Caenophidia – *Xenotyphlops* or *Amphiglossus*	–	100	100
22	30.9	*Xenotyphlops grandidieri* – *Amphiglossus* sp.	Madagascar	100	100
23	57.2	Caenophidia – *Amblyomma* or *Cornufer*	–	100	100
24	34.6	*Amblyomma limbatum* – *Cornufer pelewensis*	Oceania	100	100
25	36.8	*Hypsiscopus plumbea* – *Megophrys*	East Asia	73	100
26	37.5	Elapidae – Colubridae	East Asia	9	NS
27	11.8	*Helicops* cf. *angulatus* – *Hydrophis spiralis*	Central–South America	16	NS
28	28.8	Colubridae – Elapidae	East Asia	0	Unresolved (polytomy)
29	12.6	*Contia tenuis* – *Ophiophagus hannah*	North America	3	Unresolved (polytomy)
30	1.3	*Elaphe climacophora* – Cosmocerca simile	East Asia	61	97
31	25.8	Elapidae – Viperidae	East Asia	0	–
32	9.0	Viperidae – *Afrotyphlops* sp.	Africa	74	100
33	15.3	*Crotalus horridus* – *Psammodynastes* or *Natrix* or Elapidae	North America	5	94
34	11.8	*Psammodynastes pulverulentus* – *Natrix* or Elapidae	East Asia	7	97
35	8.7	*Natrix tessellata* – Elapidae	Europe	22	96
36	17.2	*Mimophis* cf. *mahfalensis* – *Indotyphlops* or *Hylarana*	Western–Central–Southern Asia	100	100
37	11.2	*Mimophis* cf. *mahfalensis* – *Cimex lectularius*	Western–Central–Southern Asia	84	100
38	9.6	*Indotyphlops braminus* – *Hylarana* cf. *tytleri*	Western–Central–Southern Asia	100	100
39	37.1	Pseudoxyrhophiidae – *Mantella*	Madagascar	96	100
40	29.2	*Alluaudina bellyi* – *Heterixalus* or *Guibemantis*	Madagascar	46	100
41	25.8	*Heterixalus* – *Guibemantis*	Madagascar	43	Unresolved
42	5.7	*Heterixalus betsileo* – Raillietnema sp. (ZCRK_016, 017)	Madagascar	97	Unresolved (polytomy)
43	5.6	*Madagascarophis colubrinus* – *Acrantophis* cf. *dumerili*	Madagascar	78	100
44	30.5	Pseudoxyrhophiidae – Cophylinae	Madagascar	7	Unresolved (polytomy)
45	13.9	*Cophyla* – *Gephyromantis boulengeri*	Madagascar	77	100
46	28.7	Pseudoxyrhophiidae – *Madatyphlops* or *Sanzinia* or *Phelsuma*	Madagascar	6	Unresolved (polytomy)
47	18.1	*Madatyphlops arenarius* – *Sanzinia* or *Phelsuma*	Madagascar	65	100
48	2.5	*Sanzinia* cf. *madagascariensis* – *Phelsuma lineata*	Madagascar	100	100
49	27.7	Pseudoxyrhophiidae – Common ancestor of *Boophis* & *Mantidactylus*	Madagascar	3	Unresolved (polytomy)
50	21.8	Pseudoxyrhophiidae – *Blommersia blommersae*	Madagascar	42	NS
51	2.1	*Liophidium torquatum* – Trombiculidae sp. 1	Madagascar	100	100
52	6.9	*Mantidactylus lugubris* – Raillietnema sp. (ZCRK_047)	Madagascar	100	100
53	6.9	*Boophis madagascariensis* – *Malagabdella vagans*	Madagascar	99	100
54	4.1	*Boophis madagascariensis* – *Ptychadena mascareniensis*	Madagascar	84	100

The node numbers correspond to supplementary fig. 9, Supplementary Material online. BPs, bootstrap probabilities in ML phylogeny; BPPs, bayesian posterior probabilities in BI phylogeny.

Supplementary Table 7. Models and parameters used in the geographic area estimation of HT occurrences.

Model	Parameters	*d*	*e*	*j*	lnL	AIC
DEC	2	0.01	0.01	–	-670.60	1345.21
DEC+J	3	5.5×10^{-5}	1.0×10^{-12}	0.034	-342.96	691.91
DIVALIKE	2	0.01	0.01	–	-642.39	1288.79
DIVALIKE+J	3	7.0×10^{-5}	1.0×10^{-12}	0.034	-342.32	690.63
BAYAREALIKE	2	0.01	0.01	–	-801.79	1607.58
BAYAREALIKE+J	3	4.8×10^{-5}	1.0×10^{-7}	0.034	-346.66	699.33

d, rate of range expansion by adding an area; *e*, rate of range reduction through extirpation in an area; *j*, relative per-event weight of jump dispersal at cladogenesis. lnL, log-likelihood; AIC, Akaike Information Criterion.

Supplementary References

Bossuyt F, Milinkovitch C. 2000. Convergent adaptive radiations in Madagascan and Asian ranid frogs reveal covariation between larval and adult traits. *Proc Natl Acad Sci USA*. 97(12):6585–6590.

Burbrink FT, Lawson R, Slowinski JB. 2000. Mitochondrial DNA phylogeography of the polytypic North American rat snake (*Elaphe obsoleta*): a critique of the subspecies concept. *Evolution* 54(6):2107–2118.

Gilbert C, Chateigner A, Ernenwein L, Barbe V, Bézier A, Herniou EA, Cordaux R. 2014. Population genomics supports baculoviruses as vectors of horizontal transfer of insect transposons. *Nat Commun.* 5:3348.

Ivancevic AM, Kortschak RD, Bertozzi T, Adelson DL. 2018. Horizontal transfer of BovB and L1 retrotransposons in eukaryotes. *Genome Biol*. 19(1):85.

Kocher TD, Thomas WK, Meyer A, Edwards SV, Pääbo S, Villablanca FX, Wilson AC. 1989. Dynamics of mitochondrial DNA evolution in animals: amplification and sequencing with conserved primers. *Proc Natl Acad Sci USA*. 86(16):6196–6200.

Kumar S, Stecher G, Suleski M, Hedges SB. 2017. TimeTree: A resource for timelines, timetrees, and divergence times. *Mol Biol Evol*. 34(7):1812–1819.

Kurabayashi A, Matsui M, Belabut DM, Yong HS, Ahmad N, Sudin A, Kuramoto M, Hamidy A, Sumida M. 2011. From Antarctica or Asia? New colonization scenario for Australian-New Guinean narrow mouth toads suggested from the findings on a mysterious genus *Gastrophrynoides*. *BMC Evol Biol*. 11:175.

Liu H, Fu Y, Jiang D, Li G, Xie J, Cheng J, Peng Y, Ghabrial SA, Yi X. 2010. Widespread horizontal gene transfer from double-stranded RNA viruses to eukaryotic nuclear genomes. *J Virol*. 84(22):11876–11887.

Mullen GR, Oconnor BM. 2002. Mites (Acari). In: Mullen G, Durden L, editors. Medical and Veterinary Entomology. London: Academic Press. p. 449–517.

Ortiz MF, Wallau GL, Graichen DA, Loreto EL. 2015. An evaluation of the ecological relationship between *Drosophila* species and their parasitoid wasps as an opportunity for horizontal transposon transfer. *Mol Genet Genomics*. 290(1):67–78.

Puinongpo W, Singchat W, Petpradub S, Kraichak E, Nunome M, Laopichienpong N, Thongchum R, Intarasorn T, Sillapaprayoon S, Indananda C, et al. 2020. Existence of Bov-B LINE retrotransposons in snake lineages reveals recent multiple horizontal gene transfers with copy number variation. *Genes* 11(11):1241.

Smit AFA, Hubley R, Green P. 2013–2015. RepeatMasker Open-4.0. Available from: http://www.repeatmasker.org.

Walsh AM, Kortschak RD, Gardner MG, Bertozzi T, Adelson D. 2013. Widespread horizontal transfer of retrotransposons. *Proc Natl Acad Sci USA*. 110(3):1012–1016.

Zupunski V, Gubensek F, Kordis D. 2001. Evolutionary dynamics and evolutionary history in the RTE clade of non-LTR retrotransposons. *Mol Biol Evol*. 18(10):1849–1863.

Supplementary Data 1. The taxon sampling analyzed in this study.

Supplementary Data 2. The estimation of BovB copy numbers and the genomic coverages in the frog genomes using dot blot analysis.
